# Liver fatty acid binding protein FABP1 transfers substrates to cytochrome P450 4A11 for catalysis

**DOI:** 10.1016/j.jbc.2025.108168

**Published:** 2025-01-08

**Authors:** Kevin D. McCarty, F. Peter Guengerich

**Affiliations:** Department of Biochemistry, Vanderbilt University School of Medicine, Nashville, Tennessee, USA

**Keywords:** cytochrome P450, fatty acid binding protein, protein–protein interaction, fatty acid oxidation, enzyme kinetics, kinetic modeling

## Abstract

Cytochrome P450 (P450) 4A11 is a human P450 family 4 **ω**-oxidase that selectively catalyzes the hydroxylation of the terminal methyl group of fatty acids. Cytosolic lipids are the substrates for the enzyme but are considered to be primarily bound in cells by liver fatty acid binding protein (FABP1). Lipid binding to recombinant FABP1 with a fluorophore displacement assay showed substantial preference of FABP1 for ≥16-carbon fatty acids (*K*_*d*_ < 70 nM). Comparison of palmitate-binding studies revealed that FABP1 bound the lipid >100-fold more tightly than P450 4A11. Tight binding of P450 4A11 to Alexa-488 dye–labeled FABP1 was observed in fluorescence assays, and the interaction was dependent on ionic strength (*K*_*d*_ = 3–124 nM). Kinetic studies with Alexa-FABP1 indicated that the rate of protein–protein association is fast (∼2 s^−1^), and a palmitate delivery experiment suggested that substrate transfer (from FABP1 to P450) is not rate limiting. From these results, we constructed a kinetic model of the FABP1–P450 interaction and applied it to a catalytic study of FABP1 on P450 4A11 palmitate **ω**-hydroxylation, the results of which conclusively rejected the free ligand hypothesis. Our results are explained by a direct transfer model in which lipid-bound FABP1 interacts with P450 4A11, transfers the substrate, and a slower P450 conformational change follows to position the molecule in a mode for oxidation. Given the limited free lipid pool *in vivo*, interaction with FABP1 may be a dominant mechanism by which P450 4A11 accesses its substrates and may offer a novel means to target P450 4A11 activity.

Fatty acid binding proteins (FABPs) are members of the calycin superfamily of proteins that share a common β-barrel structural motif and bind hydrophobic molecules, especially long-chain fatty acids (1). The human genome includes nine FABP genes that encode functional proteins whose distribution is often tissue specific, a property by which each ortholog was initially named (*i.e.*, liver-FABP). The nomenclature has since largely changed to a numerical code (*i.e.*, FABP1) (2), reflecting the observation that multiple FABPs may be expressed in the same tissue, and that some tissues lack a unique FABP (*e.g.*, liver-FABP [FABP1] and heart-FABP [FABP3] are both expressed in the kidney (3)).

FABPs are abundant cytosolic proteins. FABP1 alone constitutes 7 to 11% of the total protein content of liver cytosol, achieving concentrations of up to 1 mM (4). Given that FABPs are known to bind fatty acids tightly (evidenced by low nanomolar *K*_*d*_ values), it is thought that most cytosolic fatty acids are FABP bound (5). This property is thought to be bifunctional in that FABPs not only maintain physiologically relevant lipid concentrations (*i.e.*, by solubilizing them) but target the molecules to organelles bearing fatty acid oxidation machinery (*i.e.*, mitochondria, peroxisomes, endoplasmic reticulum), acting as intracellular lipid chaperones (4).

Cytochrome P450 enzymes (P450s, CYPs) constitute a superfamily of 57 heme-containing monooxygenases that are crucial to the metabolism of xenobiotics as well as physiological molecules, including steroids and sterols, fatty acids and eicosanoids, and vitamins. Of the P450s that metabolize lipids, the family 4 (CYP4) enzymes are ω-hydroxylases, named for the selectivity for the terminal methyl group of medium- to long-chain fatty acids (6, 7). The CYP4 enzymes have long been of clinical interest for the biological properties of their reactions, particularly in the ω-oxidation of the C_20_ polyunsaturated fatty acid arachidonate, which yields a vasoactive eicosanoid, 20-hydroxyeicosatetraenoic acid.

Like most human P450s, the CYP4 enzymes are microsomal proteins that localize to the cytosolic face of the endoplasmic reticulum and are expressed predominantly in hepatocytes and renocytes (3). Although their substrates (cytosolic fatty acids) are thought to exist primarily as FABP complexes in cells, the issue of a CYP4–FABP interaction and substrate transfer is apparently unexplored. The ability of P450s to receive substrates from intracellular lipid-binding proteins has been demonstrated with retinoid-metabolizing P450s and retinoid—binding proteins by the Isoherranen group (P450 family 26 enzymes) (8, 9, 10) and our own laboratory (P450 27C1) (11). Recently, the Isoherranen laboratory has considered the possibility that FABP1 interacts with xenobiotic-metabolizing P450s to receive drug molecules for oxidation (12, 13). The role(s) of extracellular proteins (such as albumin) in altering the metabolism of drugs and physiological molecules by lowering the fraction of unbound molecule available is well established (14), and thus, we present the general question in the schematic shown in Figure 1.

**Figure 1:**
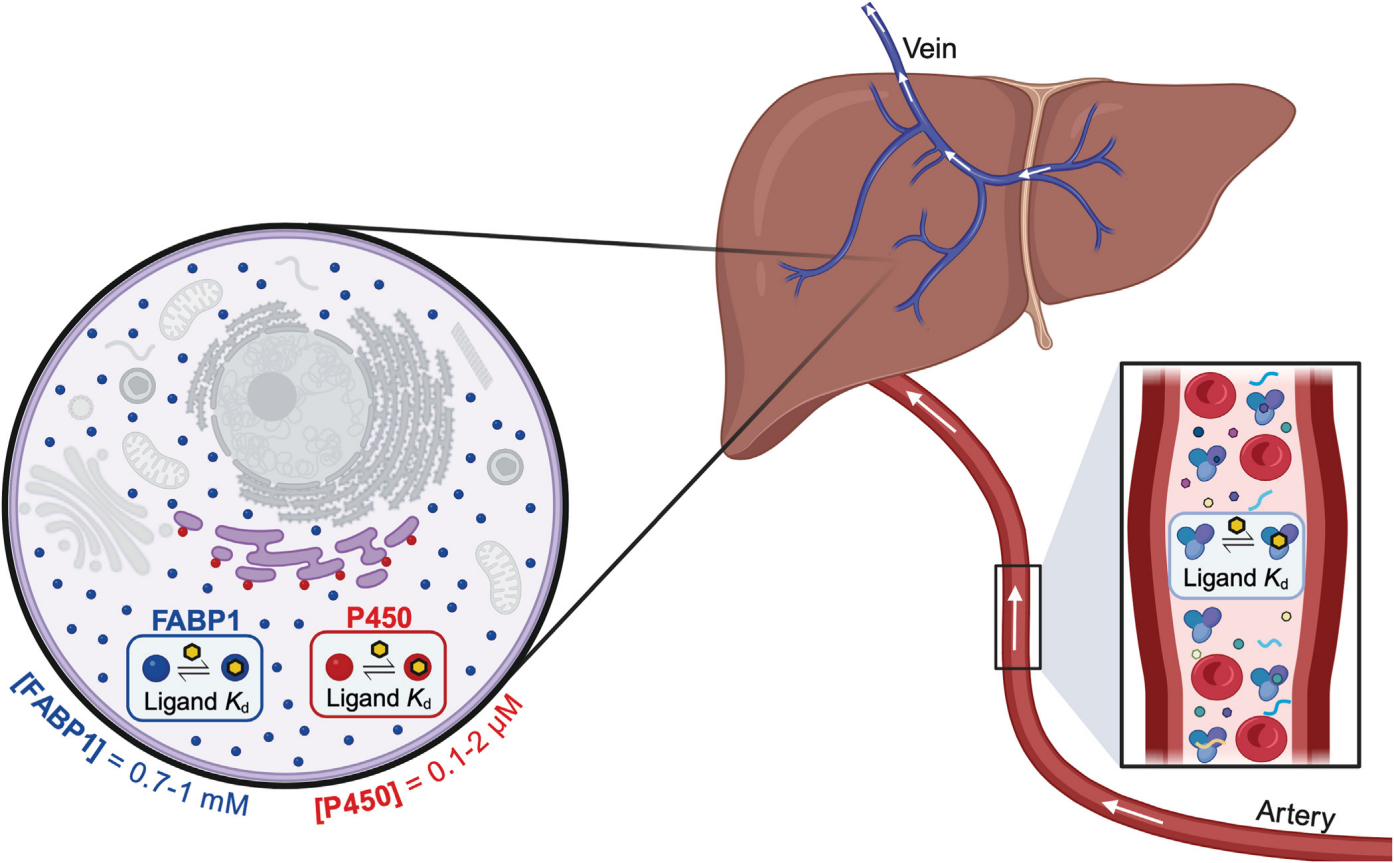
**Role of protein binding in hepatic metabolism of molecules.** The role of extracellular protein binding (*inset*, at *right*, in the bloodstream) is a common feature of hepatic (organ shown) metabolism of both exogenous and endogenous molecules. The role of intracellular (cell diagram at *left*) protein binding of the same molecules in metabolism is less well characterized. Cytoplasmic FABP1 (in *blue*) and microsomal P450 4A11 (in *red*) are indicated. Nonparticipatory subcellular organelles (*i.e.*, in the proposed P450–FABP1 interaction) are shown in *gray*. Ligand binding to proteins is labeled with “ligand *K*_*d*_.” The direction of blood flow (entry *via* “Artery,” exit *via* “Vein”) is indicated with *arrows*. Figure was made with Biorender.com. FABP1, fatty acid binding protein.

Lipids are vital constituents of physiological processes but are suspects in the pathogenesis of a number of human ailments—including diabetes, obesity, hypertension, and various metabolic syndromes (15). FABPs (for their role in lipid transport (16)) and CYP4 enzymes (for their role in lipid metabolism and eicosanoid biosynthesis) present logical drug targets in the treatment of these diseases. Small—molecule inhibitors of FABPs (15, 17) and CYP4 enzymes (18) may be useful drugs for this purpose, but lack of candidate molecules (FABPs) and issues with side effects *in vivo* (CYP4 enzymes) have hindered their development despite decades of careful study of both protein families. Identification of novel mechanisms by which to inhibit these proteins would certainly be of substantial clinical interest.

In the present work, we address the hypothesis that FABP1 transfers fatty acids to P450 4A11. A series of experiments is presented to delineate between two competing models of fatty acid binding by P450 4A11: the “free ligand” model and the “direct transfer” model (Fig. 2). The free ligand model predicts that P450 can only bind fatty acids available in solution (*i.e.*, that FABP1-bound fatty acids are sequestered from P450 catalysis), whereas the direct transfer model predicts that FABP1 interacts with P450 to deliver lipids. Herein, we have characterized fatty acid binding to recombinant FABP1 and P450 4A11 and performed steady-state kinetic and pre–steady-state experiments to discern between the two models. The FABP–P450 interaction was further probed in binding studies utilizing a fluorescent FABP1 derivative. From the results, we completed a kinetic model of the interaction and conclude that FABP1 directly transfers fatty acid substrates to P450 4A11.

**Figure 2:**
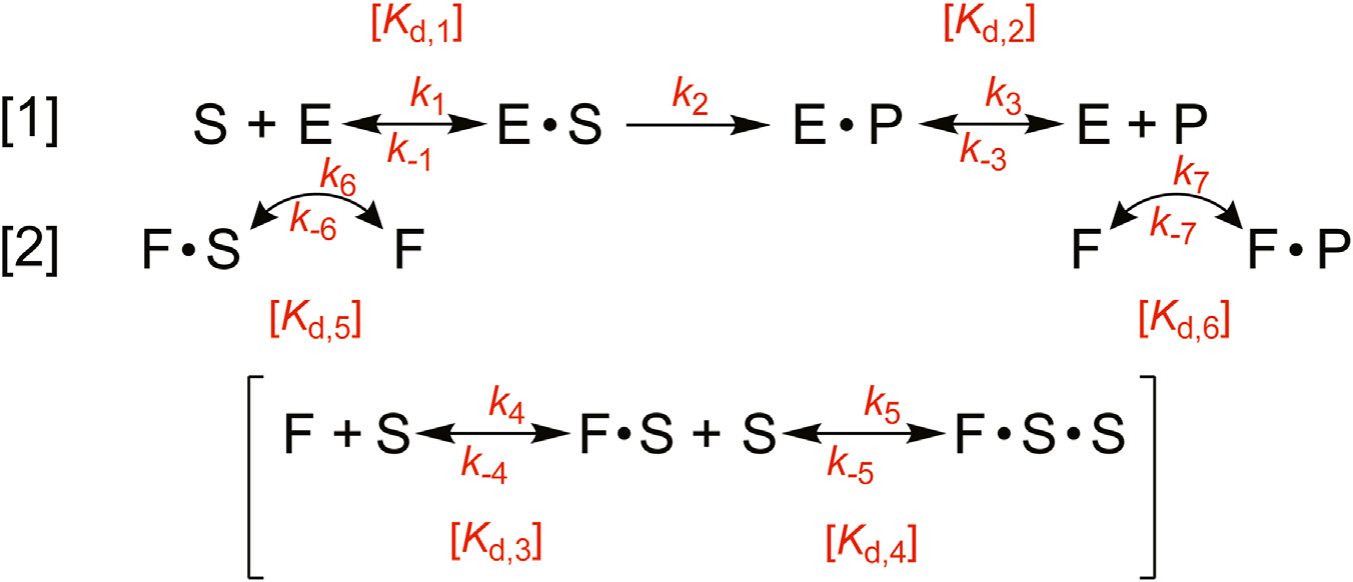
**Competing kinetic models of the role of FABP1 in P450 catalysis.** In model [1], P450 (E) binds the substrate (S) after it is released into solution by FABP1. FABP1 thereby sequesters the molecule from P450 (*i.e.*, “free ligand hypothesis”). In model [2], FABP1 (F) binds S and transfers the molecule to E to form the enzyme–substrate (ES) complex, thereby facilitating the P450 reaction. Enzymatic turnover of substate (ES) yields product (EP), which dissociates (P) and can be bound by F. All dissociation constants and rate constants (forward and reverse, where thermodynamically feasible) are labeled in *red*. FABP1, fatty acid binding protein.

## Results

### Rationale

Whether FABP1 competitively binds P450 4A11 substrates (free ligand model) or delivers them to the enzyme for metabolism (direct transfer model) is the fundamental question of the present work (Figure 1, Figure 2 and 2). To distinguish between these two competing models, we reason that the mechanism of action of FABP1 can be revealed following a kinetic characterization of both proteins, primarily considering rate constants of lipid binding and P450 catalysis and dissociation rates (Fig. 2). In the following experiments, we directly measured or estimated these kinetic parameters, *via* computational modeling or otherwise. As such, each of the indicated rates or dissociation constants presented in Figure 2 (in *red*) is addressed experimentally and corresponds to a subsection later (indicated, where appropriate, in the subheading).

### Preparation of delipidated recombinant FABP1

**Figure 3:**
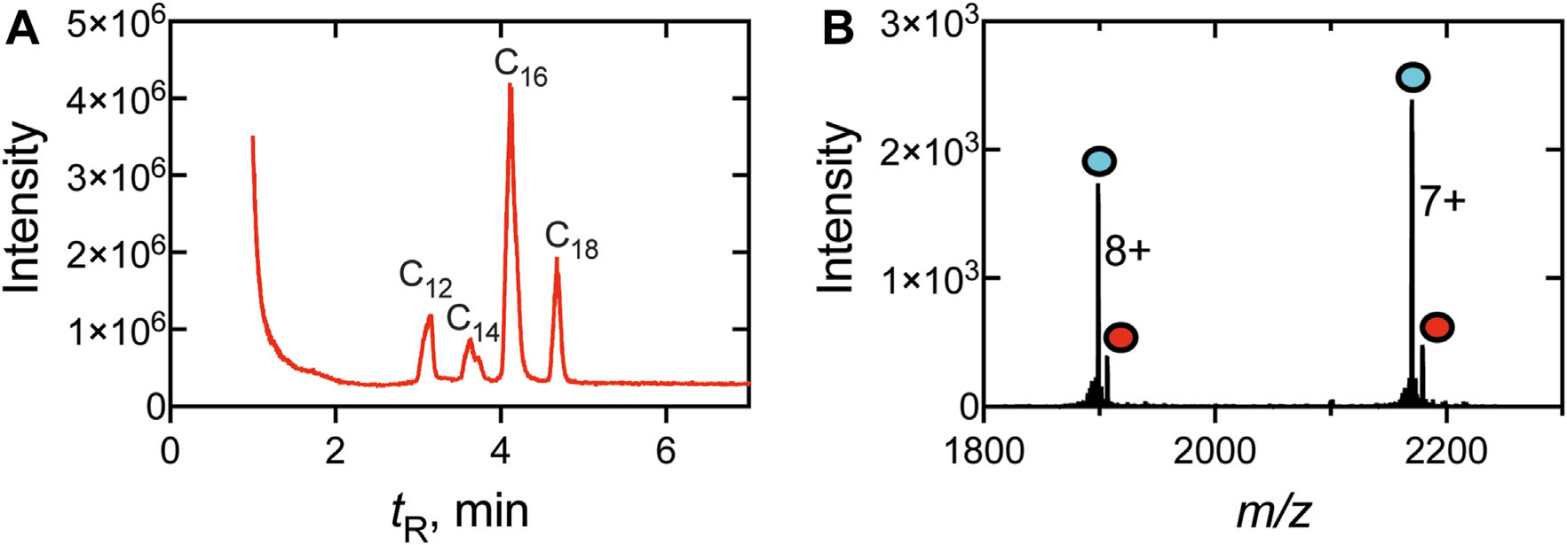
**Characterization of purified and delipidated recombinant FABP1 protein.***A,* evidence of contaminating lipids in purified FABP1 stocks (before delipidation treatment). Fatty acids were extracted from FABP1 protein solution (with hexanes), derivatized with a diazo reagent (see the Experimental procedures section), and were detected as pyridyl lipid esters by positive-ion LC–MS. Saturated fatty acids 12- to 18-carbons (C_12_ to C_18_) in length were identified (by *m/z* of their ester derivatives) as the most abundant in solution and are indicated on the total ion chromatogram trace. *B,* native mass spectrum collected with delipidated FABP1 solution (10 μM). The 8+ and 7+ charge states are labeled. Monomeric protein is indicated with a *blue sphere*, whereas protein in complex with CH_3_CO_2_H (presumably bound from the sample buffer) is indicated with a *red sphere*. FABP1, fatty acid binding protein.

A complementary DNA encoding full-length FABP1 (Fig. S1) inserted into a pGEX-69-2 vector (Fig. S2) was expressed in *Escherichia coli* DH5α cells, and the resulting FABP1 protein was purified based on published protocols (12, 19). Notably, the construct utilized N-terminal glutathione-*S*-transferase (GST) and C-terminal hexahistidine tags to facilitate initial protein purification, the former of which was removed *via* proteolytic digest to give the final protein construct used in our assays (Fig. S3). The purified protein was electrophoretically homogenous on SDS-PAGE analysis (Fig. S4), although analysis of hexane extracts of the protein stocks revealed the presence of fatty acids (Fig. 3*A*), thus indicating a mixed population of ligand-bound and ligand-free FABP1 (as had been reported previously (12)). Two successive incubations of the protein stocks with conditioned Lipidex-5000 resin were found to yield optimally delipidated (ligand-free) protein, reducing the fatty acid load on FABP1 from >5% to ≤1.2% (Fig. S5). The analysis of FABP-bound fatty acids required careful subtraction of blank samples to isolate the contribution of fatty acids that readily contaminate the laboratory environment (20, 21, 22).

The extent of delipidation was further verified by collecting native mass spectra of FABP1 stock solutions (Fig. 3*B*), where the FABP1 monomer was observed to be primarily ligand free. A significant *m/z* shift corresponding to a +60 amu ligand was reproducibly observed and was consistent with acetate binding (Fig. 3*B*), presumably bound from the buffer (see the *Experimental procedures* section). In agreement with LC–MS analysis of CH_2_Cl_2_ extracts, the native mass spectrometry (MS) data did not indicate that lipid-bound FABP1 was present at levels >1% of the total protein stock, confirming the reliability of our delipidation method. The FABP1 stocks were then quantified *via* two sensitive independent protein quantitation methods to ensure accuracy: absorbance at 280 nm (using theoretically estimated extinction coefficient; Figs. S6, S7) and *via* free cysteine (thiol) using Ellman’s reagent (see the *Experimental procedures* section). Both methods provided protein concentration values that were within 5% of each other, giving confidence in the result.

### Measurement of FABP1 dissociation constants (*K*_*d*,3_, *K*_*d*,4_, and *K*_*d*,6_)

Substrate binding to FABPs is routinely assessed *via* the ability of the potential substrate to displace a fluorophore that is bound in the FABP1 active site (23, 24). Our study utilized 11-dansylaminoundecanoic acid (DAUDA) as the fluorophore. Synthesis of DAUDA was achieved *via* a three-step Gabriel procedure (Fig. S8) in good overall yield (75%) and high purity (Figs. S9–S11). In contrast to an earlier literature report (25), we found that esterification of the fatty acid was absolutely necessary in our synthesis (for solubility in CH_2_Cl_2_) prior to the reaction with dansyl chloride.

**Figure 4:**
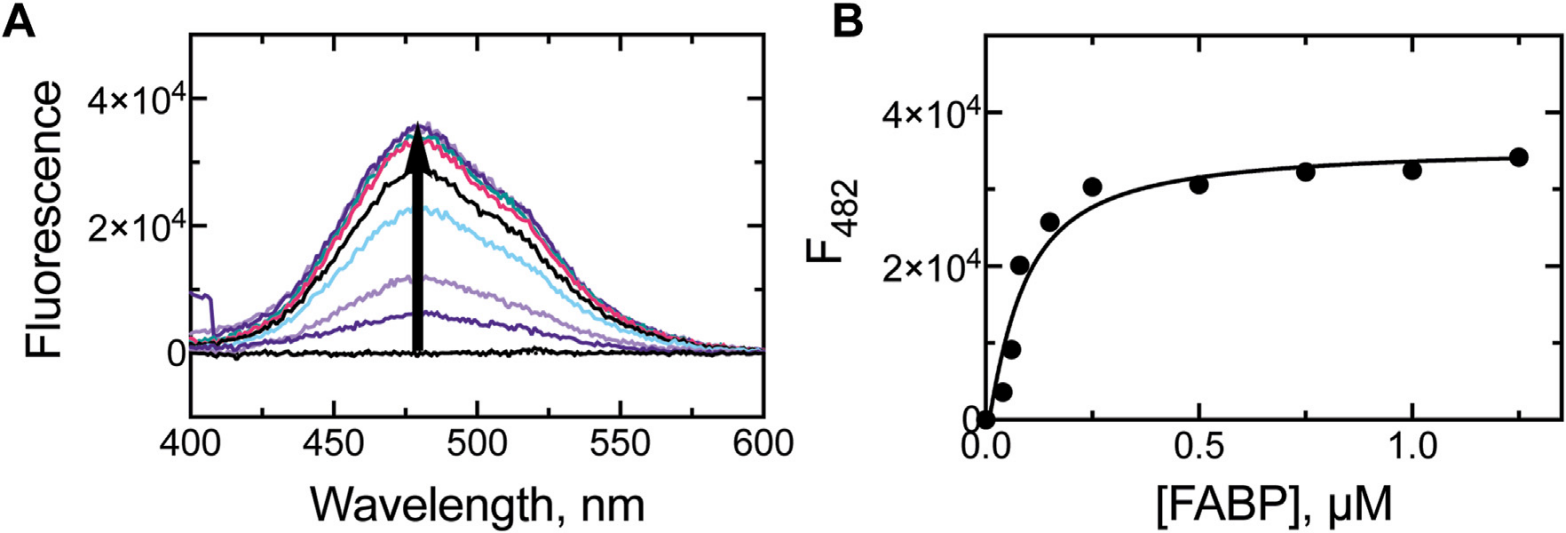
**Fluorescence titration of DAUDA with FABP1 (“reverse” titration).***A,* fluorescence spectra (λ_ex_ = 335 nm) of DAUDA (0.05 μM) collected with increasing concentrations of FABP1 (0–1.25 μM). The binding of DAUDA to FABP1 is accompanied by a gain of fluorescence, indicated with an *arrow*. *B,* titration curve calculated with data points (n = 1) at the observed fluorescence maximum (482 nm) and plotted *versus* concentration of FABP1. Data were fit to a quadratic equation. DAUDA, 11-dansylaminoundecanoic acid; FABP1, fatty acid binding protein.

Binding of DAUDA to FABP1 was assessed in a “reverse titration” where FABP1 was titrated into a fixed concentration of DAUDA (50 nM), yielding a characteristic gain of fluorescence (Fig. 4*A*) at 482 nm. FABP1 was titrated into a solution of DAUDA (“reverse titration”) and not *vice versa* because of the intrinsic fluorescence of DAUDA (Fig. S11*E*), which overlaps with the spectrum of DAUDA–FABP1 (Fig. 4*A*). The intrinsic fluorescence of DAUDA was generally only problematic at concentrations much higher (>500 nM) than used in the assays (50 nM), though we sought to eliminate this additional variable by performing the reverse titration to estimate the *K*_*d*_. When FABP1 was titrated into DAUDA, quadratic fitting revealed a *K*_*d*_ of 56 ± 23 nM(*K*_*d*,3_) for the protein–ligand interaction (Fig. 4*B*).

**Figure 5:**
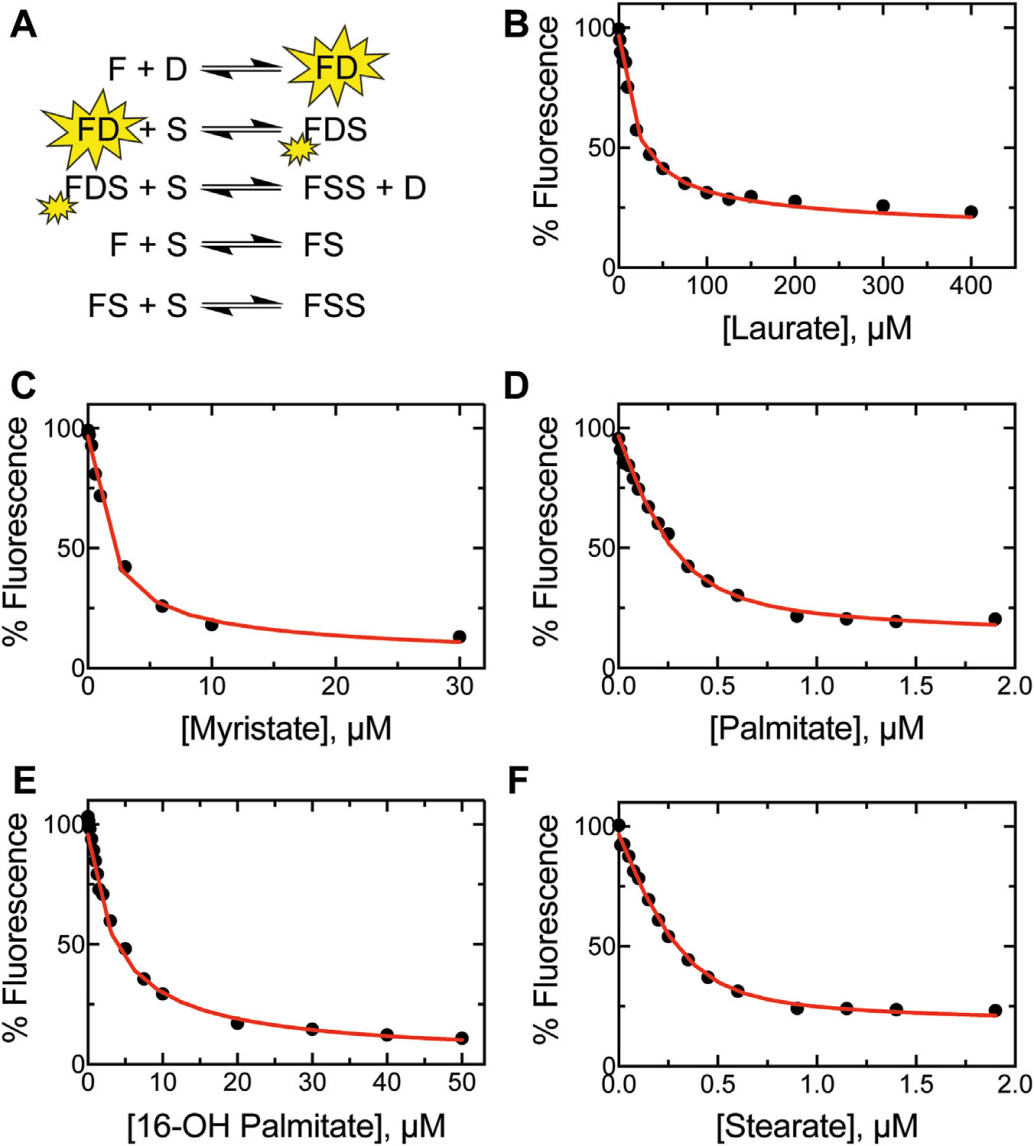
**Fluorescence titrations of DAUDA–FABP1 with fatty acids (“forward” titrations).***A*, model of all binding equilibria accounted for in the DAUDA displacement assays. In the fluorescence assays (λ_ex_ = 335 nm), DAUDA (D) binds to FABP1 (F) to form a fluorescent (indicated with *yellow flash*) complex (FD), with which a lipid (S) is then competed against for binding. The binding of S attenuates the fluorescence (denoted with a smaller *yellow flash*) and forms a ternary complex (FDS). High concentrations of S may completely displace D to form FSS, a spectroscopically silent complex. Using this method, fluorescence titrations were carried out with the fatty acids (*B*) laurate, (*C*) myristate, (*D*) palmitate, (*E*) 16-hydroxypalmitate, and (*F*) stearate. Fluorescence at the emission maximum (482 nm) was monitored (as in Fig. 4) and normalized to the starting fluorescence (*i.e.*, the FD complex prior to the addition of S). The data were fit using KinTek Explorer and are shown in Table 1. Each data point corresponds to one independent replicate. DAUDA, 11-dansylaminoundecanoic acid; FABP1, fatty acid binding protein.

The FABP–DAUDA system was used to screen saturated fatty acids (C_12_ to C_18_) for binding to FABP, and the *K*_*d*_ values were estimated in competition with the fluorophore (using its known *K*_*d*_) with the kinetic modeling software KinTek Explorer. The general model is shown in Fig. 5*A*, where the substrate is competed against the fluorescent DAUDA–FABP1 complex for binding, yielding a ternary DAUDA–FABP1–substrate complex that is accompanied by an attenuation in fluorescence. While our model includes the possibility that the substrate completely displaces DAUDA (*i.e.*, FDS + S —> FSS + D), it did not appear that DAUDA was completely displaced by any substrate at the tested concentrations.

The kinetic modeling revealed *K*_*d*_ value estimates for lipid binding to apo-FABP1 with laurate (C_12_), myristate (C_14_), palmitate (C_16_) (*K*_*d*,4_), 16-OH palmitate (*K*_*d*,6_) (the product of palmitate oxidation by P450 4A11), and stearate (C_18_) of 30 μM, 2.6 μM, 0.046 μM, 0.52 μM, and 0.069 μM, respectively (Fig. 5, Table 1). *K*_*d*_ value estimates for lipid binding to DAUDA-bound FABP1 (*i.e.*, DAUDA displacement) were 18 μM, 0.86 μM, 0.010 μM, 15 μM, and 0.021 μM, respectively, whereas estimates for binding to a ternary complex (full displacement of DAUDA) were >400 μM, 43 μM, 10 μM, 63 μM, and 34 μM, respectively (Table 1). (Data processing for FABP1 titration with palmitate and the subsequent kinetic modeling in KinTek Explorer are provided in Figs. S12 and S13). The off-rates extracted from computational modeling were used to estimate the *K*_*d*_ values listed in Table 1. Based on the findings of these experiments, palmitate was selected as the lipid substrate for further experiments with FABP1 as the molecule was observed to bind tightly to the protein.

**Table 1:** Dissociation constant (*K*_*d*_ values) estimated for the binding of lipids to FABP1

Lipid	F + S ↔ FS		FD + S ↔ FDS		FDS + S ↔ FSS + D	
	*k*_off_ (s^−1^)	*K*_*d*_ (μM)	*k*_off_ (s^−1^)	*K*_*d*_ (μM)	*k*_off_ (s^−1^)	*K*_*d*_ (μM)
Laurate (C_12_)	*2700*	30	*410*	18	*>100,000*	>1000
Myristate (C_14_)	*240*	2.6	*20*	0.86	*980*	43
Palmitate (C_16_)	*4.1*	0.046	*0.24*	0.010	*240*	10
16-OH Palmitate (C_16_)	*47*	0.52	*350*	15	*1500*	63
Stearate (C_18_)	*6.2*	0.069	*0.48*	0.021	*770*	34

Dissociation constants were estimated for lipid (S) binding (Fig. 5) to apo-FABP1 (F), DAUDA-bound FABP1 (FD), and to a ternary complex (FDS, to form FSS). Values were estimated *via* kinetic modeling as described in the *Experimental procedures* section, using on-rates of 9.0 × 10^7^ M^−1^ s^−1^ (single molecule present) or 2.3 × 10^7^ M^−1^ s^−1^ (multiple molecules present) and a DAUDA *K*_*d*_ value of 56 nM. Values in *italics* were extracted from kinetic modeling of experimentally gathered data. All values are shown to only two significant digits.

### Kinetics of DAUDA binding to FABP1 (*k*_4_)

**Figure 6:**
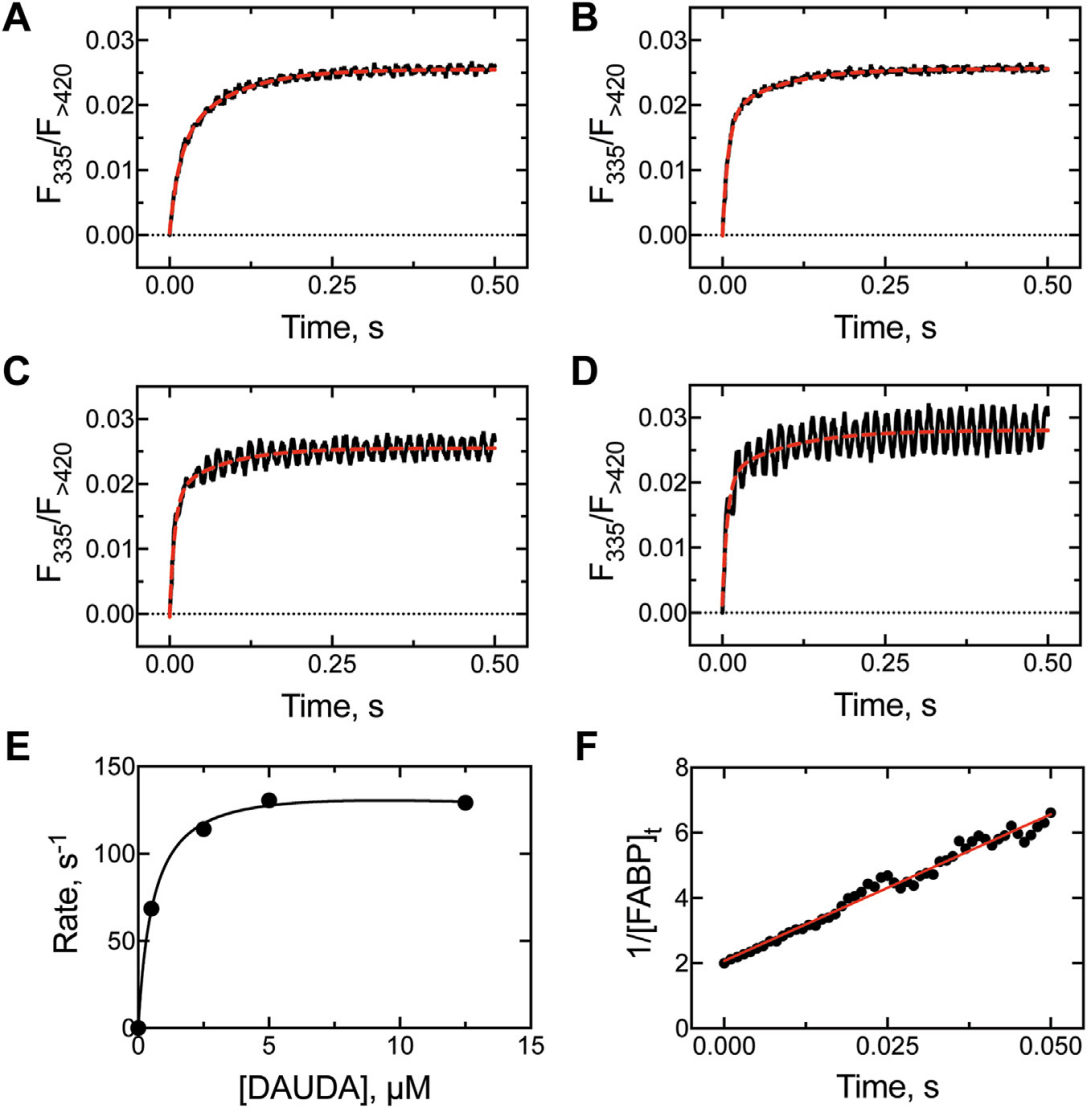
**Kinetics of binding of DAUDA to FABP1.** FABP1 (0.5 μM) was mixed with (*A*) 0.5 μM, (*B*) 2.5 μM, (*C*) 5 μM, and (*D*) 12.5 μM DAUDA to yield the binding traces shown. DAUDA–FABP1 was excited (λ_ex_ = 335 nm) as in Fig. 4 and total fluorescence signal >420 nm was collected. Data were fit to double exponential curves, and *(E*) the rate of the fast phase was extracted and plotted against DAUDA concentration. The data curve was fit to a Michaelis–Menten-type hyperbola. Each trace corresponds to ≥6 averaged replicate traces. In part *A*, the binding of DAUDA (0.5 μM) was proportional to the disappearance of protein (0.5 μM). The reciprocal concentration of FABP1 was plotted against time in part *F*, and the slope of the linear portion (linear regression in *red*) of that curve (77) gave an on-rate (*k*_on_) of 9.0 × 10^7^ M^−1^ s^−1^. DAUDA, 11-dansylaminoundecanoic acid; FABP1, fatty acid binding protein.

In order to measure the rate of binding of DAUDA to FABP1, four concentrations (0.5–12.5 μM) of DAUDA were rapidly mixed with FABP1 (0.5 μM), and the gain of fluorescence (at 482 nm, Fig. 4*A*) was monitored over time (Fig. 6, *A*–*D*). The data were fit to double exponential curves, and the rate of the initial binding step was observed to be saturable under the tested conditions (Fig. 6*E*). A *k*_on_ value (*k*_4_) of 9.0 ± 0.2 × 10^7^ M^−1^ s^−1^ was calculated from the data (see the *Experimental procedures* section) (Fig. 6*F*).

### Kinetics of palmitate binding to DAUDA–FABP1 (*k*_5_)

**Figure 7:**
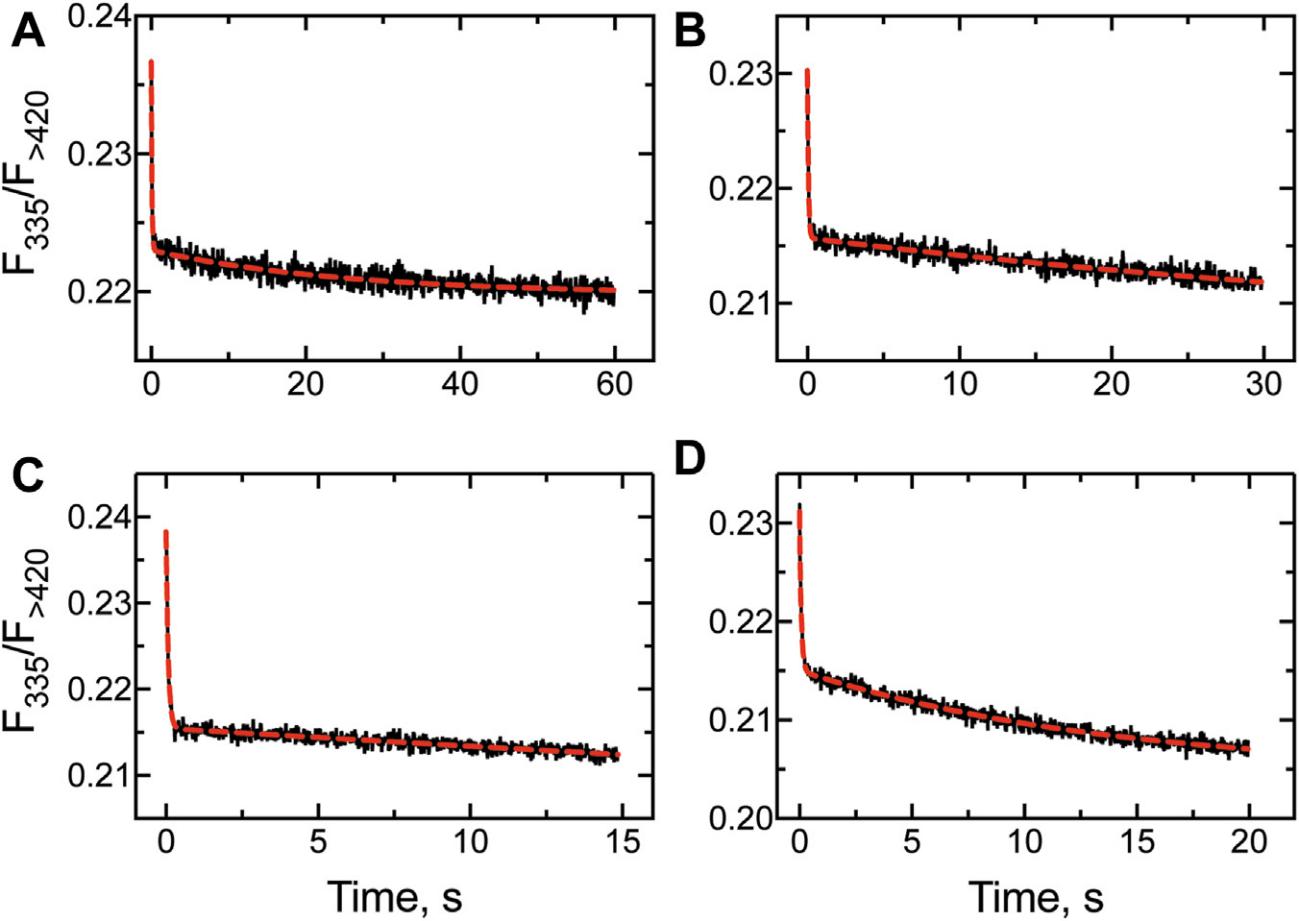
**Kinetics of binding of palmitate to FABP1**. A stoichiometric mixture of DAUDA and FABP1 (0.5 μM) was rapidly combined with (*A*) 0.5 μM, (*B*) 5 μM, (*C*) 12.5 μM, and (*D*) 25 μM palmitate, and the attenuation of fluorescence (λ_ex_ = 335 nm, λ_em_ > 420 nm) was monitored over time (as FD → FDS, Fig. 5*A*). The plots were fit to double exponential equations, and the following rate constants for each phase were extracted: *A*, *k*_1_ 11 ± 1 s^−1^, *k*_2_ 0.04 ± 0.003 s^−1^; *B*, *k*_1_ 16± 1s^−1^, *k*_2_ 0.017 ± 0.004 s^−1^; *C, k*_1_ 14 ± 1 s^−1^, *k*_2_ 0.05 ± 0.01 s^−1^; *D, k*_1_ 13 ± 1 s^−1^, *k*_2_ 0.07 ± 0.01 s^−1^. Each trace corresponds to ≥6 averaged replicate traces. DAUDA, 11-dansylaminoundecanoic acid; FABP1, fatty acid binding protein.

As mentioned previously, palmitate was selected to measure binding kinetics to DAUDA–FABP1 because of the low *K*_*d*_ observed with the protein (Fig. 5*D*, Table 1). When a ligand binds to DAUDA-bound FABP1, DAUDA is displaced (potentially to the second ligand-binding site, forming a ternary complex; Figure 2, Figure 5 and Figure 2, Figure 5*A*) and attenuates the fluorescence of the system (Fig. 5). Taking advantage of this phenomenon, we preincubated FABP1 (0.5 μM) with DAUDA (0.5 μM) to form the fluorescent complex. This complex was then rapidly mixed with palmitate (0.5 μM to 25 μM), and the attenuation of fluorescence was monitored as palmitate binds and displaces DAUDA. The data were fit to double exponential equations, and the rate of initial binding was not observed to vary with palmitate concentration (Fig. 7). A *k*_on_ value (*k*_5_) of 2.3 ± 0.2 × 10^7^ M^−1^ s^−1^ was calculated from the data (Fig. 7*A*).

### Palmitate and 16-OH palmitate binding affinity to P450 4A11 (*K*_*d*,1_, *K*_*d*,2_)

**Figure 8:**
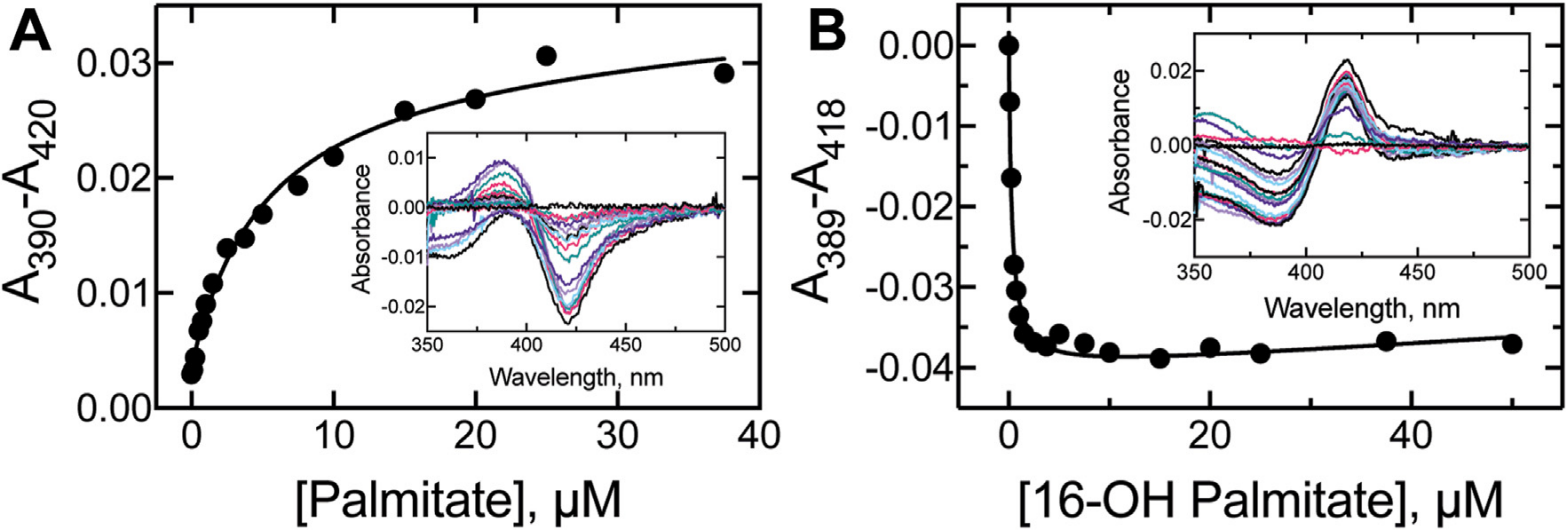
**Equilibrium binding titrations of P450 4A11 with fatty acids**. P450 4A11 (1 μM) was titrated with (*A*) palmitate and (*B*) 16-OH palmitate in a 1.0 cm pathlength cell. Difference plots were calculated from absorbance maxima (∼390 nm) and minima (∼420 nm), and the data were fit to a hyperbolic equation. The *insets* in each panel are the difference spectra between a cuvette that received the lipid substrate and a cuvette that received solvent alone. Each data point corresponds to one independent replicate.

Affinity of palmitate and 16-OH palmitate for P450 4A11 (1 μM) was assessed by monitoring the blue shift (from ∼420 nm to ∼390 nm) of the P450 heme Soret band as a substrate binds and displaces the sixth axial heme ligand (H_2_O) to yield pentacoordinated heme (“type I” shift) (26). Palmitate induced a classical type I spectral shift with P450 4A11 (Fig. 8*A*, *inset*), and hyperbolic fitting of the data gave a *K*_*d*_ value of 5.2 ± 1.6 μM (Fig. 8*A*). 16-OH palmitate induced a reverse type I shift (absorbance increase at ∼420 nm, Fig. 8*B*, *inset*), and quadratic fitting yielded a *K*_*d*_ value of 0.27 ± 0.04 μM (Fig. 8*B*).

### Kinetics of palmitate and 16-OH palmitate binding to P450 4A11 (*k*_1_, *k-*_1_, and *k-*_3_)

**Figure 9:**
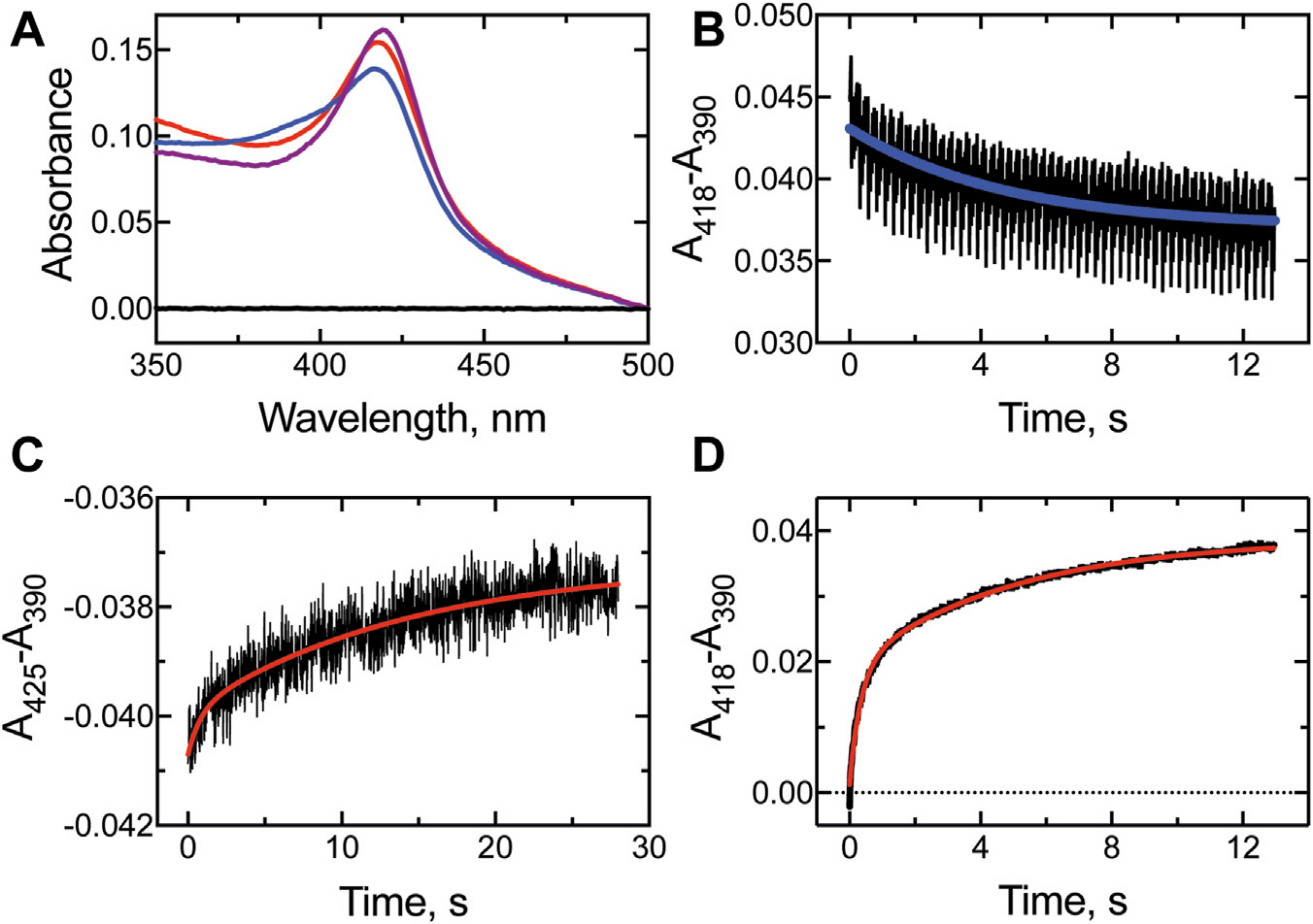
**Kinetics of fatty acid binding to P450 4A11.***A,* spectra were collected with 1 μM P450 4A11 (*purple line*) to which 5 μM palmitate (*blue line*) and 50 μM ketoconazole (*red line*) were successively added to induce characteristic P450 “type I” and “type II” spectral shifts, respectively. *B,* P450 4A11 (1 μM) was rapidly mixed with palmitate (5 μM), and the P450 blue shift (*A*) was monitored and fit to an exponential equation (*blue line*) yielding a *k*_on_ value of 0.21 ± 0.03 s^−1^. *C,* 16-OH palmitate (4 μM) and *D,* palmitate (10 μM) were preincubated with P450 4A11 (2 μM) and rapidly mixed with ketoconazole (100 μM), and the P450 red shift (part *A*) was monitored and fit to biexponential equations (*red lines*). The extracted rate constants were: *C*, *k*_1_ 1.3 ± 0.4 s^−1^, *k*_2_ 0.070 ± 0.006 s^−1^; *D*, *k*_1_ 2.9 ± 0.1 s^−1^, *k*_2_ 0.20 ± 0.01 s^−1^. Each trace corresponds to ≥6 averaged replicate traces.

To assess the rates of lipid binding to P450 4A11 (*k*_1_), type I spectral shift (from ∼420 nm to ∼390 nm, described previously) was monitored after rapid mixing of P450 and palmitate (Fig. 9*A*, *magenta* [substrate free], *blue* [substrate bound]). When P450 4A11 (1 μM) was mixed with palmitate (5 μM), slow binding kinetics were observed (Fig. 9*B*). Single exponential fitting of the data yielded a binding rate of 0.21 ± 0.03 s^−1^ (*k*_on_ = *k*_1_ = 4.2 ± 0.6 × 10^4^ M^−1^ s^−1^).

To assess the rates of lipid egress from P450 4A11 (*k-*_1_, *k-*_3_), lipids were tested in conjunction with the general P450 inhibitor ketoconazole in a “trap experiment” as we have recently described to estimate *k*_off_ rates for P450–sterol complexes (27, 28). P450–lipid complexes were rapidly mixed with ketoconazole, and the spectral shift from 390 nm (substrate bound, type I) to 418 nm (inhibitor bound, type II) P450 was monitored over time (Fig. 9*A*, progression from *blue* to *red curve*). This spectral shift arises from the direct interaction of the azole nitrogen lone pair with the heme iron (forming a hexacoordinated heme species). This experiment is performed with an excess of ketoconazole (100 μM) to facilitate rapid capture of unliganded P450 yielded at the moment of lipid dissociation, such that ketoconazole *k*_on_ approximates the lipid *k*_off_.

When P450 4A11 (2 μM) preincubated with 16-OH palmitate (4 μM, Fig. 9*C*) or palmitate (10 μM, Fig. 9*D*) was mixed with ketoconazole (100 μM), both curves displayed biphasic kinetics (as expected). (The concentration of lipid added during preincubation reflects the affinity of the complex determined in equilibrium binding titrations; Fig. 8). The first phase of each trace relates to the ketoconazole capture of the unliganded enzyme (the majority–but not all–P450 was substrate bound at equilibrium), and the second phase correlates to the capture of enzyme yielded by the egress of the bound lipid (*i.e.*, estimate of *k*_off_). Biexponential fitting revealed similar rates for the first phase of both the 16-OH palmitate (1.3 ± 0.4 s^−1^) and palmitate (2.9 ± 0.1 s^−1^), as expected (*i.e.*, capture of unliganded enzyme by ketoconazole should proceed at a similar rate in both experiments). The rates extracted for the second phase (*k*_off_) were 0.070 ± 0.006 s^−1^ (*k-*_3_) and 0.20 ± 0.01 s^−1^ (*k-*_1_), respectively. The *A*_418_ and *A*_390_ *versus* time traces used to prepare Fig. 9*D* are provided in Fig. S14.

### Binding affinity and kinetics of Alexa-FABP1 to P450 4A11 (*K*_*d*,5_)

To determine if FABP1 interacts with P450 4A11 (Fig. 2), a fluorescently labeled FABP1 protein was prepared for use in protein–protein interaction studies. We utilized Alexa-488 C5 maleimide to prepare Alexa-488-labeled FABP1 (Fig. S15), because of our previous experience with the fluorophore (29, 30) and given the fact that FABP1 has only one cysteine residue (Cys-69, which corresponds to Cys-71 in our construct; Fig. S3), which appears to be solvent exposed and located in proximity to the ligand-binding pocket (Fig. S16). Consequently, maleimide conjugation is favorable not only for its selectivity for Cys-71 but also for the convenience of this site for protein–protein interaction studies. Trypsin digestion of Alexa-FABP1 and LC–MS/MS analysis indicated that FABP1 labeling was highly selective for the target Cys-71 residue (as expected), with minimal background labeling of amines (see the *Experimental procedures* section, Fig. S17). Native MS supported this finding, indicating that the Alexa label was present primarily in stoichiometric unity to the protein (Fig. S18).

**Figure 10:**
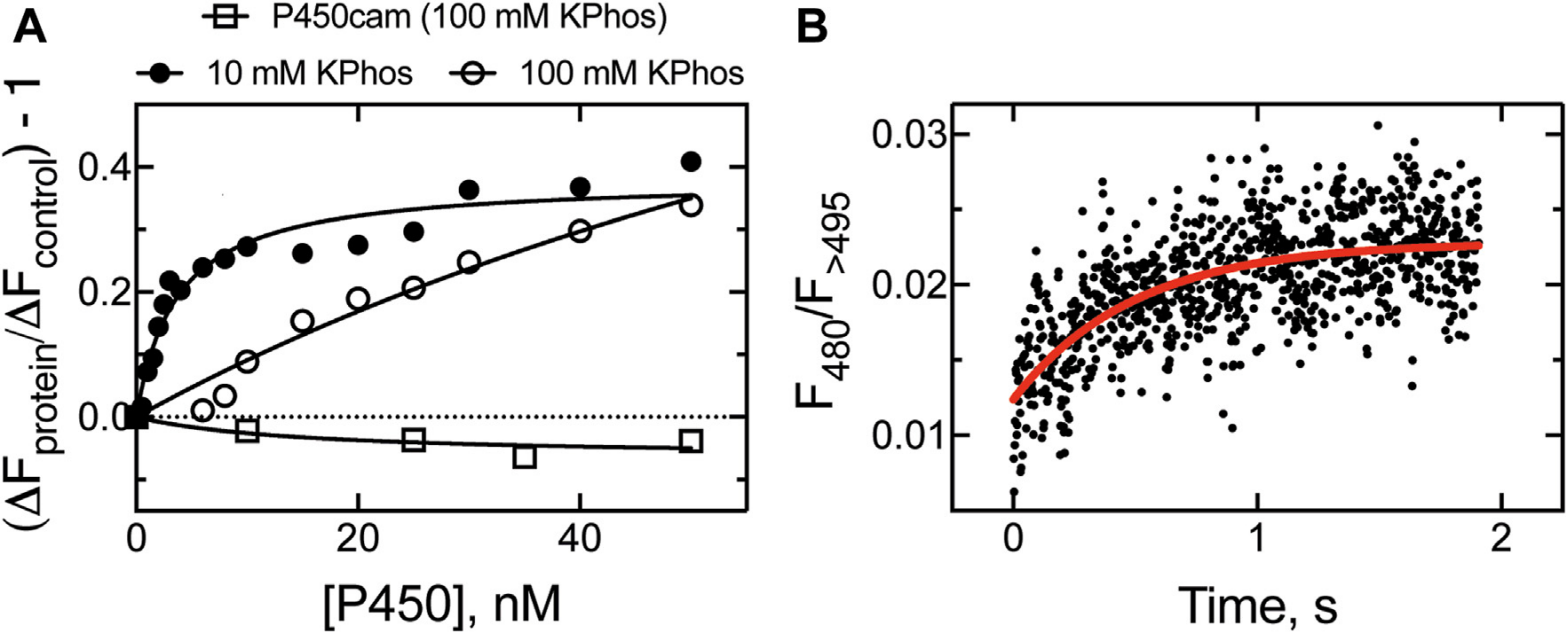
**Binding of Alexa-FABP1 to P450 4A11.***A,* equilibrium binding titration of Alexa-FABP1 (25 nM) with P450 4A11 as the titrant (0–50 nM). Titrations were performed in 10 mM (*closed circles*) or 100 mM (*open circles*) potassium phosphate buffer (pH 7.4). A control reaction was performed with P450 101A1 (P450_cam_) in 100 mM KPhos (*open squares*). The change in fluorescence upon protein–protein binding was normalized to the corresponding change in a buffer control. *K*_*d*_ = 120 ± 80 nM (100 mM, hyperbolic fit), *K*_*d*_ = 3.3 ± 0.9 nM (10 mM, quadratic fit). Each data point corresponds to one independent replicate, ≥3 biological replicates were collected to confirm the effect. *B,* kinetics of binding of Alexa-FABP1 (0.125 μM) and P450 4A11 (0.125 μM). Six traces were averaged, and the data were fit to a single exponential curve (in *red*). The extracted binding rate was 2.0 ± 0.2 s^−1^. FABP1, fatty acid binding protein.

The Alexa-FABP1 construct was tested for P450 binding in a fluorescence titration with P450 4A11. P450 4A11 (0–50 nM) was observed to enhance fluorescence upon binding to Alexa-FABP1 (25 nM), and the strength of the interaction was observed to be dependent on ionic strength of the buffer (Fig. 10*A*). At the typical buffer conditions used in our assays (100 mM potassium phosphate, Ph 7.4), the *K*_*d*_ was observed to be 120 ± 80 nM, although when the ionic strength was reduced to 10 Mm, the *K*_*d*_ decreased ∼38-fold to 3.3 ± 0.9 nM. Importantly, when a bacterial P450 101A1 (P450_cam_) was titrated into Alexa-FABP1 solution (as a control reaction), mild attenuation of fluorescence was observed; Fig. 10*A*). A similar effect was also observed when bovine serum albumin was titrated into Alexa-FABP1 (data not shown) indicating that the stimulation of fluorescence observed with P450 4A11 is unique to the human P450.

We then investigated whether the rate of protein–protein association could be estimated using our fluorescent construct. A solution of Alexa-FABP1 (125 nM final concentration) was rapidly mixed with P450 4A11 (125 Nm final concentration), and Alexa-488 fluorescence was monitored over time. The data were fit to a single exponential equation and yielded an association rate of 2.0 ± 0.2 s^−1^ (Fig. 10*B*).

### FABP1 substrate transfer kinetics to P450 4A11 (*k*_6_)

**Figure 11:**
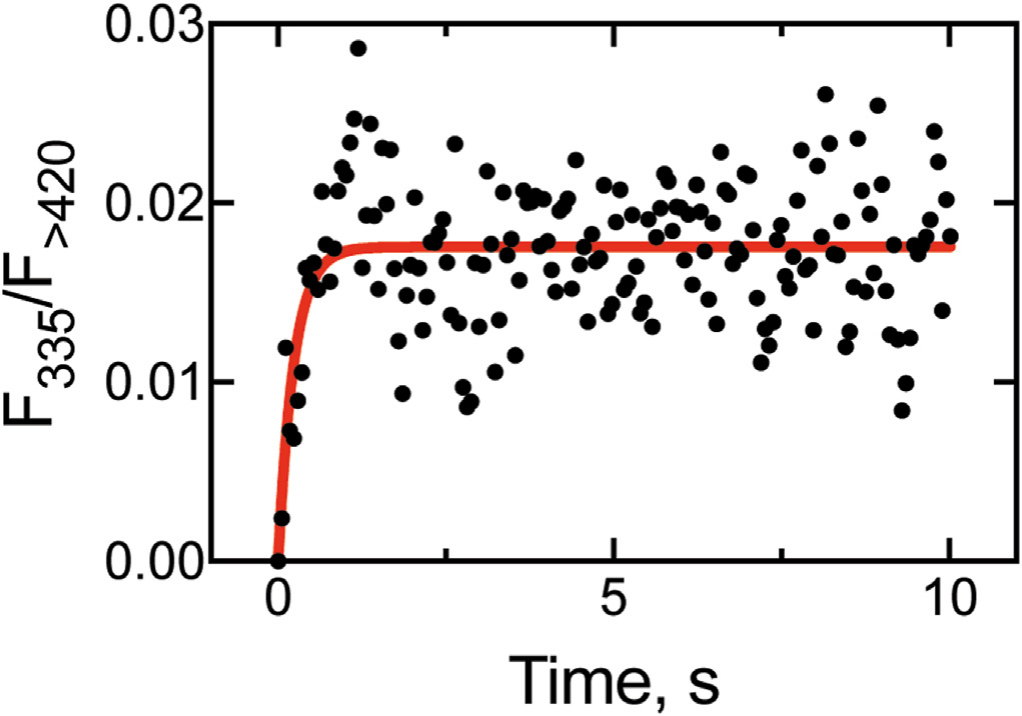
**Rate of palmitate transfer (*k***_**transfer**_**) from FABP1 to P450 4A11.** A stoichiometric mixture of FABP1, DAUDA, and palmitate (0.2 μM) was mixed with P450 4A11 (2 μM), and the gain of fluorescence (>420 nm) was monitored (*i.e.*, as FDS + E → FD + ES, Fig. 5*A*). Six traces were averaged and fit to a single exponential equation, yielding a rate of 4.2 ± 0.9 s^−1^. Seven replicate experiments were averaged to generate the trace. DAUDA, 11-dansylaminoundecanoic acid; FABP1, fatty acid binding protein.

Preliminary experiments established that P450 4A11 apparently did not bind DAUDA (Fig. S19), and we designed an experiment with the DAUDA–FABP1 system to test whether the substrate transfer rate from FABP1 to P450 could be estimated. Set up as a reverse experiment of the fluorescence displacement assay (Fig. 5), this experiment involved mixing equimolar concentrations (0.2 μM) of DAUDA, FABP1, and palmitate to form a solution (FDS↔FD+S↔FS+D) with low fluorescence (Fig. 5*A*). That solution was then rapidly mixed with an excess of P450 4A11 (2 μM), and the gain of fluorescence was monitored (*i.e.*, as substrate was transferred from FABP1 to P450, either as FDS + P450 → FD + P450-S or as FS + P450 + D → P450-S + F + D → FD; Fig. 5*A*). As expected, we observed a weak but reproducible stimulation of fluorescence in this experiment (n = 6 shots), and the data were fit to single exponential equation yielding a substrate transfer rate (*k*_6_) of 4.2 ± 0.9 s^−1^ (Fig. 11).

### Effect of FABP1 on P450 4A11 oxidation of palmitate (*k*_2_)

**Figure 12:**
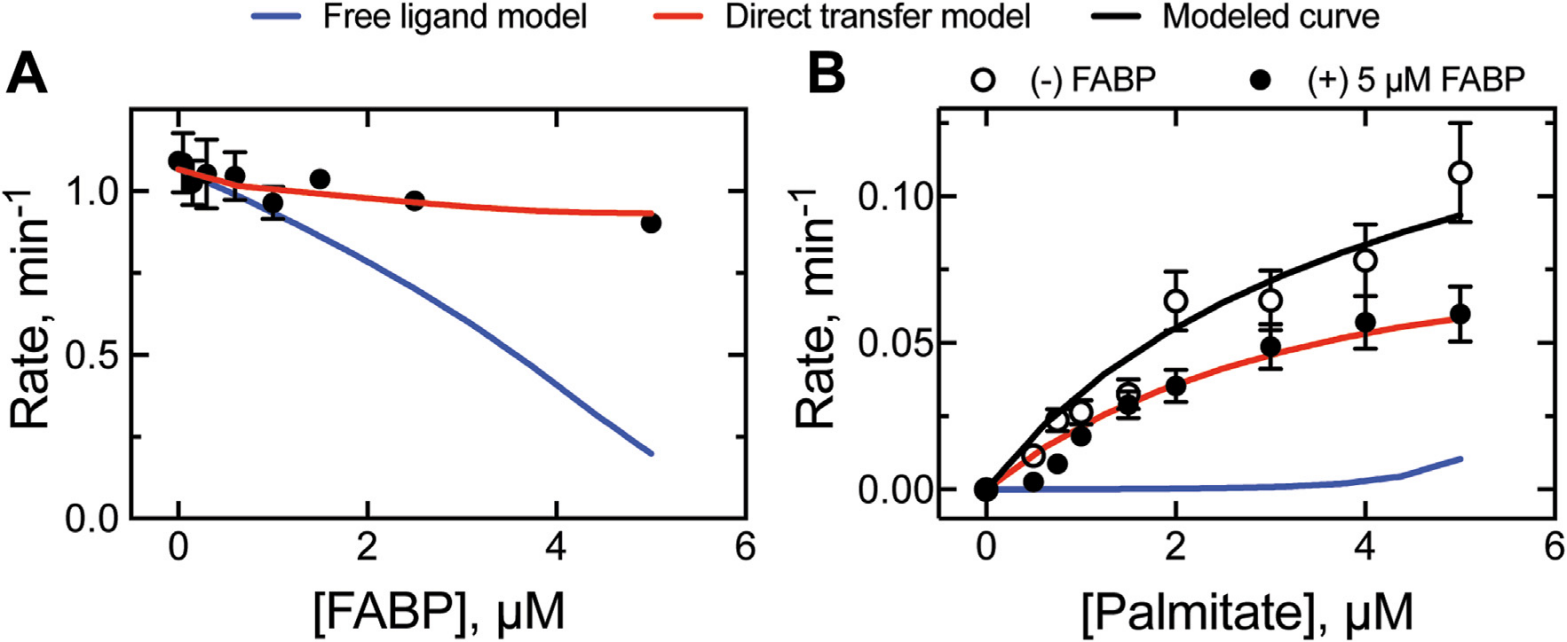
**Effect of FABP1 on palmitate ω-hydroxylation by P450 4A11.***A,* FABP1 (0–5 μM) was added to incubations (5 min) of P450 4A11 (0.15 μM) and palmitate (5 μM). *B,* palmitate (0–5 μM) was added to incubations (10 min) of FABP1 (5 μM) with P450 4A11 (0.3 μM). *Dashed lines* are the predicted kinetic data from the free ligand model (*blue*) and the direct transfer model (*red*) using the experimentally determined rate constants (Table 1, Fig. 2). A reaction performed without the addition of FABP1 (*open circles*) was also fit to a modeled curve (*black dashed line*), which utilizes only the rate constants determined for P450 4A11. Error bars reflect standard deviation of two replicate values, that is, range (if not visible, the deviation is too small to be represented). FABP1, fatty acid binding protein.

To differentiate between the two possible roles of FABP1 in P450 catalysis (competitive binder *versus* direct transfer, Fig. 2), two catalytic experiments testing palmitate oxidation were designed: one in which the FABP1 concentration was varied with a fixed concentration of palmitate (Fig. 12*A*) and another performed *vice versa* (Fig. 12*B*). When FABP1 (0–5 μM) was added to P450 4A11 reactions with palmitate (5 μM), only slight attenuation of activity was observed at the highest FABP1 concentration point tested (Fig. 12*A*). When palmitate (0–5 μM) was added to incubations containing P450 4A11 and FABP1 (5 μM), P450 4A11 activity was observed at all concentration points tested, with attenuation of reaction rate (relative to the minus FABP1 control reaction) observed at the highest substrate concentration. Preliminary experiments had established a maximum measurable rate of reaction (*k*_2_, 0.2 s^−1^), which did not saturate (Fig. S20).

### Kinetic modeling

To estimate a rate of interaction for FABP1 and P450, we loaded the protein–protein binding data (Fig. 10*B*) and fit the data to a simple model, E+F↔EF, as described in the *Experimental procedures* section. The software fit a *k*_on_ rate of 2.0 × 10^7^ M^−1^ s^−1^, close to that estimated by using the extracted binding rate ∼2 s^−1^ at the concentration (0.125 μM) of either reaction component (*i.e.*, 2 s^−1^/0.125 μM = 16 μM^−1^ s^−1^ = 1.6 × 10^7^ M^−1^ s^−1^).

To predict the potential effects of FABP1 on P450 4A11 catalysis, we programmed two computational models in KinTek Explorer to reflect those presented in Figure 2 (*i.e.*, free ligand model *versus* direct transfer model). The models were equipped with the rate constants and/or *K*_*d*_ values estimated for either palmitate or DAUDA (where applicable) and for protein–protein binding, which are summarized in Table 2. As is indicated in Table 2, certain rate constants necessary for the modeling are estimated based on a known *K*_*d*_ value and an individual known rate constant. To predict the kinetic result of the free ligand model, the rate(s) of transfer (*k*_6_, *k*_-6_) were set to a value of zero, reflecting the predicted inability of the two proteins to interact. The interaction of P450 and FABP1 occurs in the absence of substrate, although whether the P450–FABP1 (EF) complex can accept a substrate (to form EFS) is unknown. The modeling software estimated a 1 µM *K*_d_ value for the interaction with the assumption that the complex could not accept a cytosolic substrate. To predict the kinetic result of the direct transfer model, our experimentally estimated rates of transfer were allowed to be fit by the model. Aside from the manipulations with the transfer rate, all remaining rate constants were input as they appear in Table 2.

**Table 2:** Kinetic parameters utilized for reaction modeling

Protein(s)	Ligand	*k*_on_ (M^−1^ s^−1^)	*k*_off_ (s^−1^)	*K*_*d*_ (μM)
P450 4A11	Palmitate	4.2 ± 0.6 × 10^4^ (*k*_1_)	0.20 ± 0.01 (*k*_-1_)	5.2 ± 1.6 (*K*_*d*,1_)
P450 4A11	16-OH Palmitate	**2.6 × 10**^5^ (*k*_3_)	0.070 ± 0.006 (*k*_-3_)	0.27 ± 0.04 (*K*_*d*,2_)
FABP1	DAUDA	9.0 ± 0.2 × 10^7^ (*k*_4_)	**5.0**	0.056 ± 0.023
FABP1	Palmitate	9.0 ± 0.2 × 10^7^ (*k*_4_)	**4.1** (*k*_-4_)	0.046 (*K*_*d*,3_)
FABP1–palmitate–DAUDA	Palmitate	2.3 ± 0.2 × 10^7^ (*k*_5_)	*240* (*k*_-5_)	10 (*K*_*d*,4_)
FABP1, P450 4A11	N/A	*2.0 × 10*^7^ (*k*_6_)	**2.4 (k**_**-6**_**)**	0.12 ± 0.08 (*K*_*d*,5_)
FABP1	16-OH Palmitate	9.0 ± 0.2 × 10^7^ (*k*_4_, *k*_7_)	**47 (k**_**-7**_**)**	0.52 (*K*_d,6_)

All dissociation and rate constants determined in these experiments are presented (and labeled, see Fig. 2). The *k*_on_ rate of DAUDA to FABP1 was used as a proxy for the binding of one molecule of lipid to the apo-protein. The *k*_on_ rate of palmitate to DAUDA–FABP1 was used as a proxy for the *k*_on_ rate of a second lipid to a singly bound FABP1–lipid complex. Values in plain text were determined experimentally, whereas bolded values were determined mathematically in relation to an experimentally determined rate and dissociation constant. Values in *italics* were extracted from kinetic modeling of experimentally gathered data. (The number of significant digits was limited to 2.)

When provided the experimental conditions for the kinetic assays in Figure 12, the free ligand model predicted that the rate of P450 catalysis decreases rapidly as the concentration of FABP1 approaches the concentration of substrate in the experiment (Fig. 12*A*, *blue line*), which is intuitive (given the difference in palmitate binding affinities; Table 1, Table 2 and 2). Provided the same conditions, the direct transfer model predicted a slight attenuation of P450 activity (as the concentration of FABP1 approaches the total concentration of substrate (S)), which yielded an excellent fit to our experimental data when a rate of P450 catalysis of 0.11 s^−1^ was used (Fig. 12*A*, *red line*). Notably, the modeling also required a conformational transition of the enzyme–substrate complex following substrate delivery from FABP1 (*i.e.*, ${\text{ES}}^{\prime }\to \;\text{ES}$) that was not required for binding the free ligand, using forward and reverse rates of 0.086 s^−1^ and 0.013 s^−1^, respectively. In a separate experiment, the free ligand model similarly predicted that P450 activity would not be observed when the concentration of substrate was below the concentration of FABP1 (Fig. 12*B*, *blue line*). The direct transfer model predicted a hyperbolic product distribution (for [S] < [FABP1]) and gave a satisfactory fit to the experimental data when the same rates of ${\text{ES}}^{\prime }\to \;\text{ES}$ described earlier were used, but the rate of P450 catalysis was reduced 20-fold (to 0.005 s^−1^) (Fig. 12*B*, *red line*).

## Discussion

FABP1 is an abundant protein in liver cytosol: expressed at concentrations up to ∼1 mM (4), the protein outnumbers P450 enzymes ∼350- to ∼10,000-fold in hepatocytes (based on rough estimates of typical microsomal P450 content (31)) (Fig. 1). Given that FABP1 readily binds P450 substrates—such as steroids, drugs, and fatty acids (32)—the potential for FABP1 to regulate access of P450 enzymes to their substrates is definitely of interest. We sought to evaluate whether FABP1 altered the metabolism of fatty acids by a fatty acid–oxidizing P450 enzyme, namely P450 4A11. We considered two possible physiological roles of FABP1 in the P450 reaction: that FABP1 sequesters the substrate from P450 (*i.e.*, acting as a competitive binder) or that FABP1 traffics the substrate directly to P450 (*i.e.*, acting as a reaction activator). To discriminate between these two mechanistic possibilities in P450 4A11 catalysis, we assembled a kinetic model of the interaction (Fig. 2).

Prior to initiating the protein–protein interaction studies, we encountered a strategic hurdle that had been reported by others (12, 33) in that our preparations of recombinant FABP1 were contaminated with bacterial fatty acids (presumably scavenged by the protein during heterologous expression) (Fig. S5). We also observed these same fatty acids as common laboratory contaminants (Fig. S5*A*), as had been reported by others (20, 21, 22), and consequently exercised caution in the quantitation (see the *Experimental procedures* section, Fig. S5*B*). This observation further validated the need for a so-called “delipidation” (removal of endogenous lipids) step, and we determined that the method of delipidation was consequential. In our system, we found that two successive treatments of the protein with the Lipidex resin gave the cleanest result (least residual fatty acids), as opposed to the treatment of protein stocks with 1-butanol prior to a Lipidex treatment (which had been previously reported as the optimal delipidation technique for FABP1 (12, 13)).

To begin a characterization of ligand binding to FABP1, we designed our model (Fig. 2) with existing knowledge of ligand-binding stoichiometry to the protein. FABP1 has been demonstrated to have two available ligand-binding sites *via* solution NMR experiments (34), native MS (12, 34), and X-ray crystallography (35, 36). The Sharma group proposed a model of ligand binding to FABP1 based on their crystal structures of apo and ligand-bound forms in which ligand binding triggers a protein conformational change that translocates the molecule to the inner binding cavity (which we reference as “site 1”) (35). A second molecule can then bind in the initial binding channel (the portal region, which we reference as “site 2”) or alternatively displace the bound substrate (in site 1) into the portal region to occupy its initial position. The latter is considered to be the mechanism of the fluorescence displacement assay, where DAUDA initially occupies FABP1 site 1 but is displaced when a molecule of lipid binds and occupies site 1, reducing the fluorescence of the system (12). (Note that many FABP1 studies have used the dye 1-anilino-8-naphthalene-1-sulfonate instead of DAUDA.)

Inclusion of two binding sites for fatty acids in our kinetic modeling gave a satisfactory fit to the equilibrium binding titration data in all cases, and the extracted dissociation constants demonstrated one high affinity (*K*_*d*_ ∼0.05 μM, for palmitate) and one low affinity (*K*_*d*_ = 10 μM, for palmitate) binding site (Fig. 5, Table 1). The relative affinities of the two FABP1-binding sites are supported by previous literature reports, from early work with the bovine (*K*_*d*,1_ 0.26 μM, *K*_*d*,2_ >5 μM, measured with oleate (37)) and rat (*K*_*d*,1_ ∼0.01 μM, *K*_*d*,2_ ∼0.20 μM, measured with linoleate (38)) proteins to recent work with the human ortholog (*K*_*d*,1_ 0.2 μM, *K*_*d*,2_ >3.3 μM, measured with DAUDA (12)). In each of the mentioned cases, the affinity differences between the two sites are ≥20-fold, although the difference we observed was much greater (>250-fold).

Our analysis revealed a substantial preference of FABP1 for the longer-chain fatty acids (C_16_ and C_18_) tested, as the *K*_*d*_ values increased ≥650-fold from C_12_ to C_16_ (Fig. 5). This finding is consistent with earlier studies of various human FABPs that observed lipid binding affinity increased with the lipophilicity of the molecule (39, 40). The trend is especially unsurprising in our study given that 12- and 14-carbon fatty acids are not as prevalent in human physiology as ≥16-carbon members, which are critical membrane constituents and essential dietary elements (*e.g.*, linoleate and linolenate) in humans (41). Notably, the *K*_*d*_ values determined for palmitate and stearate were consistent with those reported earlier for human FABP1 *via* a fluorescence displacement assay (38 ± 1 nM and 29 ± 2 nM, respectively (42)) and with an ADIFAB system (Acrylodan-labeled Intestinal Fatty Acid Binding Protein, 60 and 23 nM, respectively (39)). The *K*_*d*_ value obtained for DAUDA and the FABP1 high-affinity site (0.056 μM) was lower but not out of range of literature estimates (0.2 μM (12) and >0.46 μM (43)).

The binding rates (*k*_on_) of palmitate and DAUDA to FABP1 (Figure 7, Figure 8) were determined to be fast with comparatively slow off-rates (*k*_off_, estimated based on experimentally determined *k*_on_ and *K*_*d*_ values) (Table 1). The values were extracted following biexponential fitting of the data, following the proposal of the Sharma group (*vide supra*) that the initial binding of ligand to FABP1 induces a conformational rearrangement of the protein (35), suggesting that ligand-binding data to FABP1 should be biphasic. Notably, our measured *k*_on_ value for DAUDA to FABP1 (0.9 × 10^8^ M^−1^ s^−1^, Fig. 6, Table 1) was within the range of reported *k*_on_ rates estimated for C_16_ to C_20_ fatty acids binding to rat FABP1 (0.9–1.4 × 10^8^ M^−1^ s^−1^ (38)). The rate of palmitate binding (2.3 × 10^7^ M^−1^ s^−1^) to DAUDA–FABP1 was slower (Fig. 7), likely reflecting the additional time required to displace the bound ligand (the *K*_*d*_ values estimated for each ligand to the unliganded protein were nearly identical).

Compared with FABP1, palmitate binding to P450 4A11 was considerably weaker (Fig. 8) and was characterized by both considerably slower *k*_on_ and *k*_off_ rates (Fig. 9). The *k*_on_ value measured for DAUDA binding to FABP1 was >2000-fold faster than palmitate binding to P450 4A11, whereas the *k*_off_ rate was 25-fold faster (Table 2). Importantly, the *k*_off_ of palmitate from the FABP1–palmitate complex (Table 1, 4.1 s^−1^) was estimated to be significantly (10–100 fold) faster than the binding of palmitate (1–10 μM) to P450 4A11 (Table 1). This observed polarity of lipid binding and dissociation rates indicates that compared with P450 4A11, FABP1 rapidly equilibrates with substrates in solution. This simple fact limited the experiments we were able to perform to distinguish between the two possible catalytic roles of FABP1 considered in Scheme 1. For example, the use of isotopically labeled substrate (in an “isotope dilution experiment”) was convincingly used in our laboratory’s work with P450 27C1 and retinoid—binding proteins (11), wherein labeled and unlabeled substrates are simultaneously delivered to the enzyme, with the labeled substrate prebound to a binding protein. The case for targeted substrate delivery can then be made based on the isotopic distribution of the products. This experiment, however, is not feasible in the (present) case of rapid equilibration of the substrate to the binding protein (*i.e.*, the binding protein (bound to labeled substrate) will rapidly equilibrate to ∼50% labeled substrate prior to reaction initiation).

The *k*_on_ and *k*_off_ values determined for P450 4A11 and palmitate were notably slower than we had previously determined for P450 4A11 and laurate (44, 45) (Fig. 9, Table 1). Our values reported herein were determined *via* two independent methods, with the on-rate extracted from the progression of the P450 blue (type I) spectral shift and the off-rate extracted from the rate of reversal of the blue shift as an inhibitor (ketoconazole) captured the free enzyme yielded from substrate dissociation to induce a red (type II) spectral shift (Fig. 9). Importantly, the quotient of the two experimentally determined rates (as *k*_off_/*k*_on_) yields a *K*_*d*_ estimate of 4.8 μM for palmitate binding affinity, while our experimentally determined value was 5.2 μM (Fig. 8, Table 2). Given that the estimated *K*_*d*_ from our experimentally determined rate constants is within experimental error of our determined value, we are confident that our rate constants have been determined accurately.

Another curiosity in the lipid-binding results with P450 4A11 is our previous observation that the product of P450 4A11 ω-hydroxylation of laurate (12-OH laurate) induced a typical type I spectral shift (44), whereas the current study found 16-OH palmitate to clearly induce a reverse type I shift. Given that it is the interaction with the oxygen atom lone pair (of H_2_O) that forms the ∼420 nm complex (hexacoordinated, ligand-free P450), we suggest that it is the oxygen atom of the product hydroxyl moiety that interacts with heme to facilitate this atypical absorbance shift. As the active sites of CYP4 enzymes are thought to be rigid to facilitate selective ω-oxidation over the competing (energetically favorable) ω-1 reaction (which has been the leading hypothesis for many years (46, 47)), product binding may place the hydroxyl moiety in close proximity to the heme iron, supporting our hypothesis.

For FABP1 to shuttle substrates to P450 4A11, the proteins must interact. To determine if FABP1 interacts with P450 4A11, we prepared a fluorescent FABP1 derivative by labeling the protein (at Cys-71) with Alexa-Fluor 488 dye, a label we have used previously to study the interactions of P450 enzymes with their corresponding redox partners (28, 29, 30, 48). The resulting Alexa-FABP1 conjugate showed fluorescence stimulation upon addition of P450 4A11, and the interaction was observed to be sensitive to ionic strength (Fig. 10*A*), as we have observed with the interaction of P450 27C1 with its redox partner adrenodoxin (30) and P450 17A1 with the accessory hemoprotein cytochrome *b*_5_ (48). Importantly, control titrations with the bacterial protein P450_cam_ and bovine serum albumin did not show fluorescence stimulation when added to Alexa-FABP1, giving confidence that the fluorescence gain is attributed to a P450–FABP interaction.

Although we observed the tightest interaction (*K*_*d*_ ∼3 nM) at low (10 mM phosphate) ionic strength, we did not subsequently lower the buffer strength in our catalytic assays in an effort to fine-tune the interaction of the two proteins. This decision has historical precedent, as it was established in early literature with rabbit liver microsomes that low ionic strength (<100 mM phosphate) reduces the rates of P450-catalyzed oxidations (49). Studies with recombinant rat P450s 1A1 (50) and 1A2 (51) have largely supported this, finding that the optimal buffer strength for the enzymatic reactions was >50 mM and >200 mM, respectively. The dependence of P450 activity on ionic strength is most likely because of the interaction between the enzyme and NADPH–P450 reductase (POR), which is thought to be optimal at high ionic strength (as is the reduction of cytochrome *c* by the enzyme (52, 53)). While we are not aware of the exact ionic strength in a liver cell, some have suggested a physiological value to be ≥100 mM (54, 55). In line with this, and to maintain consistency with our previous work with P450 4A11 (44, 56), we maintained a higher (100 mM phosphate) ionic strength throughout all experiments, despite the weaker (*K*_*d*_ ∼0.12 μM) P450–FABP1 interaction.

We further probed the kinetics of the P450–FABP1 interaction using the Alexa-FABP1 conjugate, estimating a rate of protein–protein association of ∼2 × 10^7^ M^−1^ s^−1^ (Fig. 10*B*). The rate of protein–protein association appeared to be fast, and the overall rate of transfer of the substrate from FABP1 to P450 also appeared to be rapid (Fig. 11). While the former experiment does not account for the rate of transfer (only protein–protein binding), the rate estimate for the interaction is much greater than *k*_on_ of the free substrate (∼4.2 × 10^4^ M^−1^ s^−1^). One consistent observation in previous studies of the interaction of P450s with binding proteins has been the reduction of catalytic activity in the presence of binding protein as the rate of transfer becomes rate limiting, that is, that *k*_transfer_ < *k*_on_ of the free substrate (P450 family 26 enzymes (8, 9, 10) and P450 27C1 (11) with retinoid-binding proteins, and P450 2C9 with FABP1 (12)). Our experiments with FABP1 and P450 4A11 suggest that FABP1 *k*_transfer_ is greater than *k*_on_ for the free substrate and that transfer from FABP1 is not rate limiting. However, in the course of our kinetic modeling, we observed that a second step following substrate shuttling was required to obtain a satisfactory fit to our experimental data. We consider that while the substrate is delivered to P450 quickly, a slow conformational change is then required for the protein to accept the substrate in a catalytically competent conformation. Notably, this step was only observed when substrate was delivered to P450 from FABP1 and not when it was bound from solution. This is not surprising in light of the known complexity of the binding of free substrates to mammalian P450s and the need for conformational changes to generate catalytically productive complexes (45, 57, 58).

With our kinetic model (Fig. 2) fully programmed with the necessary rate constants (Table 2), we directed the software to predict the effects of FABP1 on product formation by P450 4A11 (Fig. 12). To predict the free ligand hypothesis model, we set the rate of substrate transfer (from FABP1 to P450) to zero (to indicate no transfer) but otherwise set the modeled parameters as they appear in Table 2. In reactions of P450 4A11 with FABP1, whether the concentration of substrate was fixed and the concentration of FABP1 was varied (Fig. 12*A*) or *vice versa* (Fig. 12*B*), the free ligand hypothesis predicted the near ablation of P450 activity when [S] < [FABP1] in both experiments. This is due in part to the considerably lower *K*_*d*_ of FABP1 for palmitate than P450 (∼0.05 μM *versus* 5.2 μM, *i.e.,* 100-fold). If the free ligand hypothesis is applied in our tested system, FABP1 would be expected to nearly completely outcompete P450 for substrate binding and thus prevent P450 catalysis (at [FABP] ≥ [S]).

Our experimental data simply did not fit the free ligand hypothesis model, as the enzyme activity was reduced in both experiments but never >50% (at the maximum concentration of FABP1 or substrate, Fig. 12). Rather, our data yielded a remarkably close fit to the direct transfer model, which was modeled from the parameters in Table 2. As mentioned, our observation that FABP1 reduced the overall *k*_cat_ of the P450 reaction is consistent with earlier studies of P450s and binding proteins (8, 9, 10, 11, 12), though we interpret the current observation as a result of a slow conformational change occurring in the enzyme after transfer from the binding protein. The kinetic modeling conclusively demonstrates that the free ligand hypothesis should be rejected, and our results can only be explained in the context of a direct protein–protein transfer model in the scenario.

The capacity of FABP1 to transfer fatty acids to model membranes has been demonstrated (59), and direct binding of FABP1 to anionic phospholipids has been reported (observed under low ionic strength) as a feature of the fatty acid transfer interaction (60). While it is conceivable that FABP1 might transfer fatty acids to microsomal membranes and that those molecules then embark on a trajectory toward a membrane-anchored P450 enzyme, this theory would not explain our experimental observations reported herein. First, our protein–protein association experiments with Alexa-488 FABP1 (Fig. 10) were performed in the absence of phospholipid, that is, the stimulation of fluorescence that we observed in the determination of *K*_*d*_ and the protein–protein association kinetics experiments could not have come from the binding of FABP1 to a membrane mimic. Second, our substrate transfer experiments with FABP1 (Fig. 11) indicated that substrate transfer from the binding protein was not rate limiting. The rate of FABP1 lipid transfer to phospholipid membranes was reported to be quite slow (generally ∼0.005 s^−1^ (59)), whereas our rate of transfer (∼5 s^−1^) was ∼1000-fold faster. Third, we utilized a simple system, consisting only of three proteins P450 4A11, POR, and FABP1, along with a minimal phospholipid vesicle component needed to bring the P450 and POR together, in a reductionist approach, avoiding more complex interactions as much as possible. (Although cytochrome *b*_5_ does stimulate the reaction about twofold [Fig. S20], it was omitted here to keep the kinetic analysis simpler.) Finally, our catalytic experiments with FABP1 were fit well to a computational kinetic model that was informed by rate constants that were determined in the absence of phospholipid, further strengthening our case for direct P450–FABP substrate transfer.

FABPs have been previously demonstrated to interact with nuclear receptors—particularly peroxisome proliferator–activated receptors (61, 62). Given that the interaction was initially identified by coimmunoprecipitation (of FABP1 and peroxisome proliferator–activated receptor), the protein–protein complex is presumably strong. With this in mind, we find our proposal of a protein–FABP1 interaction–mediating substrate transfer to have some precedent, and that our experimental design clearly eliminates an FABP1–phospholipid transfer mechanism as an alternative hypothesis for our results.

The surface of FABP1 is dominated by positive electrostatics that are thought to support the binding of anionic ligands (55). Our observation of a tight-binding salt-sensitive interaction of FABP1 with P450 4A11 may suggest an additional function of this basic surface. Interaction of P450 enzymes with redox partners is often considered to proceed *via* contact of the basic P450 surface with the acidic surface of the redox partner (48, 63). We propose that the interaction of FABP1 proceeds *via* a separate interaction surface, so as not to disrupt the interaction of P450 4A11 with NADPH–P450 reductase, requiring negative P450 electrostatics—though identification of that surface remains an ongoing effort.

There are nine protein-coding FABP genes in the human genome (1), and their distributions (at the protein level) are often tissue specific (2). The possibility for unique tissue-specific functions of these proteins has been considered and in the context of our work may suggest that interaction of FABPs with P450 enzymes may be a more general feature of lipophilic ligand binding across the enzyme superfamily.^1^ We consider that the possibility of an FABP–P450 interface may provide a novel means by which to target a P450 reaction catalyzed with an FABP-bound substrate.

## Experimental procedures

### Chemical synthesis

The three-step synthesis for DAUDA was based on an earlier report (25) but with the modification that the carboxy terminus of 11-aminoundecanoic acid was esterified for solubility during the reaction with dansyl chloride in CH_2_Cl_2_.

#### Synthesis of 11-aminoundecanoic acid ethyl ester **(1)**

To a solution of 11-aminoundecanoic acid (1.5 g, 7.4 mmol; Sigma–Aldrich) in C_2_H_5_OH (45 ml) was added H_2_SO_4_ (concentrated, 1 ml) dropwise with stirring. The resulting mixture was heated under reflux overnight. The solvent was evaporated (to a volume of ∼10 ml) and was transferred to an ice-cold water and extracted with ethyl acetate (3 × 30 ml). The organic layer (∼100 ml) was washed with saturated NaHCO_3_ solution (100 ml) (now pH ∼7–8), and brine (3 × 100 ml), dried over anhydrous Na_2_SO_4_, filtered, and evaporated. The reaction did not fully go to completion (as judged by TLC [20% CH_3_OH in CH_2_Cl_2_, v/v, product *R*_f_ 0.65]), but the resulting crude material was used without further purification (1.27 g).

#### Synthesis of 11-dansylaminoundecanoic acid ethyl ester **(2)**

To a solution of the aforementioned ester (**1**) (0.97 g, 4.4 mmol) in CH_2_Cl_2_ (50 ml) was added diisopropylethylamine (1 ml) and dansyl chloride (1 g, 3.7 mmol). The reaction mixture was stirred in the dark (covered) at room temperature (22 h). The mixture was evaporated, and the resulting material was purified by silicic acid column chromatography (5% CH_3_OH in CH_2_Cl_2_) to yield the title compound as a yellow oil (1.67 g, 95% yield).

#### Synthesis of 11-dansylaminoundecanoic acid **(3)**

To (**2**) (∼3 ml) was added 2% KOH in C_2_H_5_OH (50 ml), and the solution was heated under reflux at 60 °C for 48 h in the dark. The mixture was evaporated to ∼7 ml, chilled on ice, and neutralized with HCl (which precipitated a yellow solid). The resulting mixture was extracted with ethyl acetate (3 × 75 ml), and the organic layer was washed with brine (3 × 225 ml), dried over anhydrous Na_2_SO_4_, filtered, and evaporated to afford the title compound (**3**) as a yellow powder (1.54 g, 99% yield). ^1^H NMR (600 MHz, CDCl_3_): δ 1.08 to 1.18 (m, 8H), 1.19 to 1.25 (m, 2H), 1.26 to 1.30 (m, 2H), 1.35 (quin, 2H, *J* = 7.2 Hz), 1.60 (quin, 2H, *J* = 7.5 Hz), 2.87 to 2.90 (m, 2H), 2.89 (s, 6H), 4.78 (brs, 1H), 7.19 (d, 1H, *J* = 7.5 Hz), 7.52 (dd, 1H, *J* = 8.3, 7.5 Hz), 7.56 (dd, 1H, *J* = 8.6, 7.6 Hz), 8.25 (dd, 1H, *J* = 7.3, 0.9 Hz), 8.29 (d, 1H, *J* = 8.6 Hz), 8.54 (d, 1H, *J* = 8.5 Hz). ^13^C NMR (150 MHz, CDCl_3_): δ 24.8, 26.5, 29.0, 29.1, 29.26, 29.30, 29.6, 34.2, 43.4, 45.6, 115.3, 118.9, 123.3, 128.5, 129.8, 130.0, 130.5, 134.9, 152.1, 179.5. NMR spectra are provided in Figs S9, S10. Positive-ion electrospray LC–MS (*m/z* 435.3, Fig. S11, *A*, *C*) and LC–UV (Fig. S11, *B*, *D*) confirmed the identity and purity of the product.

#### Storage of DAUDA **(3)**

The dried powder was stored in a sealed round-bottom flask at −20 °C. For storage of DAUDA solutions, see “Stability of DAUDA stock solutions” later.

### Proteins

#### Preparation of P450 4A11 and NADPH–P450 reductase

P450 4A11 (64, 65) and NADPH–P450 reductase (66) were expressed in *E. coli* and purified as described previously. P450 101A1 (P450_cam_) was expressed and purified as described previously (67, 68). P450 concentration was determined spectrophotometrically using an OLIS-Aminco DW2a instrument (On-Line Instrument Systems) in the split-beam mode as described (69) (Fig. S21).

#### Preparation of liver FABP (FABP-1)

##### Expression of FABP1

A complementary DNA encoding N-terminally GST-tagged and C-terminally His_6_-tagged full-length FABP1 (GST-FABP1) (Fig. S1) inserted into a pGEX-69-2 vector (Fig. S2) was purchased from GenScript, and an expression protocol in *E. coli* was developed based on published protocols (19). The GST-FABP1 plasmid was transformed into *E. coli* DH5α cells according to standard procedures. An aliquot of the transformation (∼100 ng plasmid DNA) was plated onto fresh LB agar supplemented with ampicillin and incubated at 37 °C overnight. A single colony was swiped from the bacterial lawn and used to inoculate LB broth (50 ml) containing ampicillin (100 μg ml^−1^) and dextrose (20 mM). The culture was incubated overnight (∼19 h, 37 °C) with shaking (200 rpm). Aliquots (5 ml) of the preculture were used to inoculate Terrific Broth media (0.5 l, in 2.8-l Fernbach flasks) containing ampicillin (100 μg ml^−1^). These bulk cultures were incubated with shaking (37 °C, 200 rpm) until the absorbance reached ∼0.9 to 1.0 at 600 nm, at which point GST-FABP1 expression was induced by the addition of IPTG (1 mM). The cultures were allowed to incubate (37 °C, 200 rpm, 6 h) until harvest by centrifugation (4000*g*, 25 min, 4 °C). Cell pellets were stored at −80 °C until purification.

##### Purification of FABP1

All purification steps were performed at 4 °C unless otherwise noted. Cell pellets were thawed on ice and gently resuspended in sonication buffer (50 ml/cell pellet derived from 1 l) composed of PBS (1×, pH 7.3), PMSF (1 mM), and one cOmplete EDTA-free Protease Inhibitor Cocktail tablet (Roche) per 50 ml resuspension. The resuspension was sonicated using a probe sonicator (20 cycles [20 s on/40 s off], 45% intensity) in a stainless-steel beaker (on ice) with rotation (about the probe, for stirring). The lysate (a viscous wheat-colored homogenate) was centrifuged (14,000*g*, 30 min, 4 °C) to remove debris, and the supernatant was incubated (3 h, 4 °C, with end-over-end rotation) with nickel–nitrilotriaceticacid (Ni–NTA) resin (10 ml, in a 50-ml conical tube) that was washed with H_2_O (10 column volumes) and pre-equilibrated in equilibration buffer (1× PBS [pH 7.3], 10 column volumes). The Ni–NTA beads were collected *via* centrifugation (1000*g*, 5 min, 4 °C) and added to a gravity flow column and were washed with equilibration buffer (12 column volumes) containing 30 mM imidazole. GST-FABP1 was then eluted with equilibration buffer (five column volumes) containing 300 mM imidazole, and the eluate was reduced with DTT (1 mM) and concentrated (4000*g*, 4 °C) using 10 kDa molecular weight cutoff (MWCO) centrifugal concentrators (GST-FABP1≈ 41 kDa). The sample (<1.5 ml) was applied to a 5 ml HiTrap (GE Healthcare) desalting (gel filtration, exclusion limit ∼5 kDa) column (according to the manufacturer’s protocol) that was equilibrated in GSTrap buffer A (1× PBS [pH 7.3] containing DTT [1 mM]) to remove imidazole.

GST-FABP1 (15 ml) was applied (in 5 ml volumes) to a GSTrap HP (GE Healthcare) column (5 ml) using an FPLC (Bio-Rad) system equipped with a Superloop (50 ml sample capacity), *A*_280_ monitor, and automated fraction collector. The GSTrap column had been pre-equilibrated (five column volumes, 5 ml min^−1^) with GSTrap buffer A. The protein solution (5 ml) was loaded (0.1 ml min^−1^), and the column was washed with buffer A (five column volumes, 5 ml min^−1^). GST-FABP1 was then eluted (three column volumes, 5 ml min^−1^) with Tris buffer (50 mM, pH 8.0) containing NaCl (150 mM), DTT (1 mM), and reduced glutathione (GSH, 10 mM). GST-FABP1-containing fractions were pooled and concentrated to <1.5 ml using 10 K MWCO centrifugal concentrators (4000*g*, 4 °C) and applied to a HiTrap desalting column (as described earlier) equilibrated in Digest Buffer (Tris buffer [50 mM, pH 7.5] containing NaCl [150 mM], EDTA [1 mM], and DTT [1 mM]) to remove GSH. The FABP1-containing fractions were pooled and diluted twofold (to ∼5 ml), and the concentration of GST-FABP1 was estimated (using *ε*_280_ = 20,400 M^−1^ cm^−1^ predicted by the ExPASY ProtParam tool (70) for the fusion, Fig. S6) based on an absorbance at 280 nm (Fig. S7). To cleave the GST tag from GST-FABP1, approximately ∼1 unit of PreScission Protease (GenScript) was added per milligram of protein estimated at this stage, and the mixture was incubated (7 h, 4 °C) to achieve maximal digestion.

To collect FABP1 (and remove cleaved GST), the digest was added to Ni–NTA resin (10 ml) and pre-equilibrated in Ni–NTA buffer #2 (100 mM potassium phosphate buffer containing NaCl [100 mM] and 2-mercaptoethanol [2 mM]), rocked with end-over-end rotation as described previously (3 h, 4 °C), and the Ni–NTA beads were collected *via* centrifugation (1000*g*, 5 min, 4 °C). The Ni–NTA resin was added to a gravity flow column and was washed with Wash Buffer (Ni–NTA buffer #2 + 30 mM imidazole, 12 column volumes); FABP1 was then eluted with elution buffer (Ni–NTA buffer #2 + 300 mM imidazole, five column volumes). The elutate was pooled (∼50 ml), reduced with DTT (1 mM), and concentrated (4000*g*, 4 °C) using 3 K MWCO centrifugal concentrators (FABP1 ∼15 kDa, Figs. S3, S6). The FABP1 solution (<1.5 ml) was desalted using a HiTrap desalting column (as described earlier) to remove imidazole.

The sample was reapplied to a GSTrap column (as described earlier) to remove any undigested GST-tagged FABP1. The column (5 ml) was equilibrated in 100 mM potassium phosphate buffer (pH 7.4) containing NaCl (100 mM) (*i.e.*, buffer A). The conditions (buffer B composition, flow rates, etc.) were unchanged from the first GSTrap column. The desired FABP1 protein eluted in the column load because it does not have a GST tag, and only the uncleaved GST-FABP1 binds to the column. The FABP1 was collected in the flow-through fraction (in buffer A), and an SDS-PAGE gel was used (Fig. S4) to confirm electrophoretic homogeneity of the protein. Concentration of the protein was determined (always in the absence of DTT) using a bicinchoninic acid assay, *A*_280_ determination (Figs. S6, S7), and Ellman’s reagent (71). The latter two assays were found to give protein quantitation values that were within 6% of one another, whereas the bicinchoninic acid assay gave estimates within ∼20% of either method. For these reasons, we are confident in our FABP1 quantitation and generally used the *A*_280_ quantitation method.

##### Delipidation of FABP1

To remove lipids from FABP1 protein stocks, a protocol was developed based on an earlier report that optimized a delipidation protocol for human FABP1 (12). For the delipidation, Lipidex-5000 (a lipophilic Sephadex derivative with an average alkoxy group chain length of 15 carbons) was purchased from Revvity. Optimal delipidation of FABP1 had been reportedly observed after 3× incubations (extractions) of the protein stocks with 1-butanol followed by one incubation of the protein with Lipidex-5000 (0.1 g Lipidex-5000 per milligram protein).

Prior to incubation with FABP1 (in delipidation experiments), Lipidex-5000 resin was conditioned to swell in buffer. Lipidex-5000 resin (∼4 g) was added to a 30 ml glass vial (avoiding plastic) and centrifuged (500*g*, 5 min, 23 °C) to pellet the resin. The solvent was decanted, and FABP1 buffer (100 mM potassium phosphate buffer containing 100 mM NaCl) was supplemented with 20% CH_3_OH (v/v)—20 ml was used to resuspend the resin *via* gentle stirring with a Pasteur pipette. The resin was incubated in a bath sonicator for 10 min and was pelleted *via* centrifugation (as before). The solvent was decanted, the pellet was resuspended (by stirring) in 20 ml of FABP1 buffer (devoid of CH_3_OH), the mixture was sonicated as before (10 min), and the process was repeated for three total sonication rounds. The resin was stored overnight with light rocking (23 °C), and the majority (all but ∼1 ml per gram Lipidex) of the solvent was removed prior to use.

Three trial delipidation experiments were performed in which 40 nmol of FABP1 (in FABP buffer) was subjected to (i) 1× incubation (30 min, 23 °C) with Lipidex-5000, (ii) 2× incubations (30 min, 23 °C) with Lipidex-5000, or (iii) 3× extractions of the protein solution with an equal volume of 1-butanol followed by 1× incubation (30 min, 23 °C) with Lipidex-5000. All incubations done with Lipidex-5000 were performed using 0.1 g Lipidex-5000 per milligram protein, and at the end of the incubation, the resin was separated from the protein solution with the use of a gravity flow column housing. To assess the effectiveness of delipidation (the lipid load on the protein), we extracted and derivatized the bound lipids and analyzed them using an LC–MS approach as described earlier (See “Quantitation of ligand-bound FABP1” mentioned previously). Results are presented in Fig. S5.

#### Preparation of Alexa Fluor 488–labeled FABP1 (Alexa-FABP1)

(The target of the maleimide conjugation is the Cys-69 residue (Fig. S16) [Cys-71 in our construct, Fig. S3]). Alexa Fluor 488–labeled FABP1 (Alexa-FABP1) was prepared by adapting previously published protocols for the preparation of Alexa Fluor 488–labeled human adrenoxodin (30) and cytochrome *b*_5_ (29, 48). Briefly, Alexa Fluor 488 C5 maleimide (Invitrogen) (dissolved in dimethyl sulfoxide to 10 mM) was added (never >10%, v/v) to 2 × 15 nmol aliquots of FABP1 (that had been reduced with DTT [10 mM], incubated [30 min, 23 °C], and then desalted [with a HiTrap column, see the aforementioned purification of FABP1] to remove excess DTT) in molar ratios of 1:5 and 1:20. Excess dye (and dimethyl sulfoxide) was removed *via* a HiTrap desalting column. The presence of bound Alexa Fluor to the protein was verified by collecting the absorbance and fluorescence spectra (Fig. S15). The labeling efficiency was confirmed *via* analysis of FABP1 peptides (Fig. S17) and native MS (Fig. S18) (see aforementioned corresponding sections). The 20:1 stoichiometry Alexa-Fluor incubation was found to yield better labeling of FABP1, and the conjugate prepared using that treatment was used in all subsequent assays.

### Analysis of FABP1

#### Quantitation of ligand-bound FABP1

FABP1 (10 nmol, quantified by *A*_280_ measurements, Fig. S7) was diluted (to 0.5 ml) in H_2_O, acidified (to pH <1) with HCl, and extracted with hexanes (3 × 2 ml). The extracts were pooled and brought to dryness under a stream of N_2_. The derivatization of the lipids was based on a protocol using a diazo reagent reported earlier (to form pyridyl ester derivatives readily ionizable on positive ion electrospray LC–MS) (72) with a derivatization protocol that was further optimized later (73). The dried residue was dissolved in *tert*-butyl methyl ether (2 ml) to which CH_3_OH was added (0.2 ml) to facilitate the derivatization. The diazo reagent was added (0.25 ml, ∼1 mM) and allowed to react (10 min, 23 °C). The solvent was removed under a N_2_ stream, and the pyridyl lipid ester derivatives were dissolved in 0.2 ml CH_3_OH, transferred to autosampler vials, and analyzed by LC–MS.

Samples (10 μl, held at 25 °C) were injected on a 2.1 mm × 100 mm (1.7 μm) Acquity BEH octadecylsilane (C_18_) column (held at 40 °C) using a Waters Acquity UPLC with a flow rate of 0.3 ml min^−1^ and a gradient mobile phase of (A) 5% aqueous CH_3_CN (10 mM ammonium acetate) and (B) CH_3_OH (10 mM NH_4_OAc) as follows (all % v/v): 0 min, 85% B; 0.5 min, 85% B; 1.5 min, 100% B; 5.5 min, 100% B; 5.6 min, 85% B; 7 min, 85% B. The column eluate was subjected to electrospray ionization, and products (pyridyl fatty acid esters) were detected using a Waters QDa on-line mass spectrometer (Waters) operating in the positive-ion mode using a cone voltage of 15 V, a sampling frequency of 10 Hz, and scanning from *m/z* 150 to 600. Data were processed using MassLynx software (Waters) and are presented in Fig. S5.

#### Analysis of FABP1 peptides

Samples of FABP1 (10 μM) and Alexa-FABP1 (10 μM) were prepared for peptide-level analysis *via* S-trap (Protifi) using the manufacturer’s recommended protocol including reduction of the sample with DTT (5 mM) and alkylation with iodoacetamide (10 mM). The resulting peptide digests were dissolved in a solution of aqueous HCO_2_H (0.2% [v/v]) for analysis by LC coupled with tandem MS (LC–MS/MS). A reversed phase analytical column (∼20 cm) was packed (in-house) with C_18_ packing material (Jupiter, 3 μm beads, 300 Å; Phenomenex) directly into a laser-pulled emitter tip. A Dionex Ultimate 3000 nanoLC and autosampler was used to load peptides onto the analytical column (360 μm outer diameter × 100 μm inner diamater) using a mobile phase of (A) 0.1% HCO_2_H (aqueous) and (B) 0.1% HCO_2_H in CH_3_CN. The column was eluted at a flow rate of 350 nl min^−1^ using a gradient mobile phase as follows (all %B [v/v]: 0 min, 2%; 64 min, 40%; 71 min, 95%; 72 min, 2%; 80 min, 2%). Peptides were analyzed on an Orbitrap Exploris 240 mass spectrometer (Thermo Fisher Scientific), equipped with a nanoelectrospray ionization source. The MS/MS instrument method and the data processing (*via* MSFragger [FragPipe, version 19.1] search of tandem mass spectra) for peptide identification remained unchanged from previously reported (48). The Alexa-488 modification was primarily observed on Cys-71 (Fig. S17), though a low background labeling of free amines (lysine residues and the protein N terminus) was also observed. The labeling of lysine residues appeared to be minimal, though was not quantifiable as modification of lysine residues results in a missed cleavage by the trypsin protease, yielding lysine-labeled Alexa-488 peptides that were much longer than their unmodified counterparts. Differences in ionization efficiency between these peptides prevents the direct comparison of their intensities.

#### Analysis of FABP1 *via* native MS

FABP1 samples were concentrated on centrifugal concentrators (3 K MWCO, as described earlier) and were diluted, concentrated, and washed with ammonium acetate buffer (0.5 M) four times (*i.e.*, reducing the concentration of potassium phosphate and NaCl from 100 mM to <50 nM). Approximately ∼2 μl of FABP1 solution (∼8 μM) was loaded into borosilicate glass emitters (manually pulled in-house from glass capillaries), and a platinum wire was inserted into the solution. FABP1 ions were generated *via* nanoelectrospray with a 1 kv spray voltage potential 100 ms accumulation time from 500 to 10,000 *m/z* mass window using a ThermoFisher UHMR mass spectrometer. Native mass spectra were processed (FABP1 is a 15,185.4 Da protein; Fig. S4) using Xcalibur Qualbrowser (ThermoFisher Scientific) software (version 2.0.7).

### Preparation and quantitation of DAUDA stock solutions

#### Preparation of DAUDA stock solutions

An initial stock solution of DAUDA was prepared (∼10 mM, in C_2_H_5_OH) initially by weighing out ∼3 mg on an analytical balance and dissolving the sample in an appropriate amount of solvent. An aliquot of that solution (10 μl) was then diluted to 100 μM for the concentration to be quantitated spectrophotometrically. An OLIS-Cary 14 spectrophotometer set to scan from 220 to 500 nm utilizing a UV lamp and 1 cm quartz microcuvettes (0.35 ml) was used to record a solvent baseline, after which the diluted ∼100 μM DAUDA stock solution was added to the sample cuvette. Three spectra were recorded and averaged, and an absorbance maximum was observed at 335 nm (A_335_) and a minimum (baseline) at 498 nm (A_498_) (Fig. S11*D*). To calculate the concentration of the diluted stock, we used Beer’s law, that is (*A*_335_ – *A*_498_)/(*ε*_335_) (1 cm) = 5060 M^-1^ cm^−1^ as described later. Based on the spectrophotometrically determined concentration of the (100-fold) diluted stock, we then back-calculated to the concentration of the initial stock. That stock was then diluted to 100 μM in buffer (100 mM potassium phosphate buffer [pH 7.4]) for use in assays. Where indicated (to avoid an organic solvent effect), DAUDA stocks (10 mM, then diluted to 100 μM) were prepared in 50 mM K_2_CO_3_.

#### Stability of DAUDA stock solutions

DAUDA stock solutions (10 mM, prepared in C_2_H_5_OH) could be stored at −20 °C for up to 1 week with no significant decay of the UV spectrum and no formation of degradation peaks in the LC–MS chromatogram. Storage under these conditions for more than 1 week did compromise sample integrity (and the samples were discarded). We later observed that DAUDA stock solutions (10 mM) prepared in 50 mM K_2_CO_3_ and frozen at −80 °C could undergo several freeze–thaw cycles over the course of 2 months with no significant decay of the UV spectrum and no formation of degradation peaks in the LC–MS chromatogram. Although most of our experiments were initially performed with DAUDA stock solutions prepared in organic solvent (to be consistent with earlier studies), we have found that preparation of DAUDA in K_2_CO_3_ is optimal as it reduces the potential for solvent effects (in the DAUDA displacement assays), increases the shelf life of the stock solutions, and avoids potential changes in concentration because of solvent evaporation.

#### Measurement of DAUDA molar extinction coefficient (ε_335_)

The extinction coefficient of DAUDA at 335 nm was measured using the same protocol noted previously for the measurement of the DAUDA stock solution. The appropriate volume of C_2_H_5_OH was carefully added (to 10 mM), and the stock was diluted to 100 μM as described previously. Three spectra were recorded and averaged, and the resulting *A*_335_ – A_498_ value of 0.506 for the 100 μM sample calculated an extinction coefficient (*ε*_335_) of 5060 M^−1^ cm^−1^. We used this value for all DAUDA stock calculations in place of the literature value of 4400 M^-1^ cm^−1^ (measured in CH_3_OH) (74). We also prepared DAUDA stock solutions at 10 mM in K_2_CO_3_ (50 mM) and estimated the *ε*_335_ extinction coefficient in the same way described previously (using buffer instead of organic solvent), obtaining a value of 4550 M^−1^ cm^−1^.

### *K*_*d*_ determinations

#### Titrations of FABP1 with lipids, DAUDA

Stock solutions of DAUDA (100 μM in potassium phosphate buffer [pH 7.4]) were prepared as described previously, and lipid stocks (20 mM, in C_2_H_5_OH) were diluted to the appropriate concentration range such that the final organic composition of the cuvette did not exceed 2% (v/v).

For the reverse titration (with FABP1 as the titrant), a solution of DAUDA (0.05 μM) was prepared in potassium phosphate buffer (pH 7.4) and a baseline was recorded in a 1 cm quartz cuvette (Starna Cells) using an OLIS DM-45 spectrofluorometer (1.24 mm slit width) with an excitation wavelength of 335 nm and scanning emission spectra from 400 to 600 nm.

For the titrations with lipids, solutions of FABP1 (0.5 μM) and DAUDA (0.25 μM) were prepared such that the fluorescent species was not present in excess (75). A baseline was recorded with phosphate buffer, and a starting (basis) spectrum was recorded of the FABP–DAUDA complex (1 ml). The lipid solutions were gradually added to the cuvette with gentle mixing (pipetting up and down ∼7× with a pipette tip), and spectra were recorded (Fig. S12*A*). Control titrations were also performed the same day with solvent alone (Fig. S12*B*) as a normalizing factor for the lipid titrations as fluorescence attenuation was also observed to a much lesser degree as a solvent effect (Fig. S12*C*, *D*). Fluorescence maxima were observed at 482 nm (*F*_482_), and minima were observed at 600 nm (the titration endpoint at which all spectra converged). The data were processed such that within the lipid titration and the solvent titration, the *F*_482_ values were normalized to the starting value (the initial FABP1–DAUDA complex with nothing added, set to 100%). The relative fluorescence of the lipid titration was then normalized to the relative fluorescence of the solvent titration to adjust for fluorescence attenuation observed in the solvent titration (presumably from altering the fluorophore environment). Thus, the data were processed according to $y={\left(\frac{F_{482}\;}{F482\;\left(\text{initial}\right)}\right)}_{\text{lipid}}/{\left(\frac{F_{482}\;}{F482\;\left(\text{initial}\right)}\right)}_{\text{solvent}}$, and the resulting values were plotted as a function of lipid concentration (Fig. S12*E*). The curve was imported into KinTek Explorer (76) and was fit to a kinetic model (Fig. S13) to determine the *K*_*d*_ values (Table 1). Within the KinTek software, all fluorescent species required fluorescence scaling factors to convert signal to concentration. Scaling factors for the FABP–DAUDA complex, FABP–DAUDA–lipid complex, and DAUDA alone were 460, 92, and 8, respectively.

#### Titrations of P450 4A11 with lipids

Equilibrium binding titrations with P450 4A11 (1 μM) were performed in buffer (100 mM potassium phosphate [pH 7.4]) as described previously (44, 56). Briefly, a baseline was first acquired on an OLIS-Cary 14 spectrophotometer set to scan from 350 to 500 nm in 1 cm glass cuvettes. Lipids (in C_2_H_5_OH) were gradually titrated into the sample cuvette while an equivalent amount of solvent was added to the reference cuvette, ensuring the final organic solvent composition (*i.e.*, at the endpoint of the titration) did not exceed 2% (v/v). The cuvettes were mixed with a plumper after additions, and spectra were recorded. The spectra were exported into Microsoft Excel software, and the absorbance values at 418 nm were subtracted from those at 390 nm and the values were plotted as a function of lipid concentration. The curves were fit to either to hyperbolic (for palmitate data, as *K*_*d*_ >P450 concentration) or quadratic (for 16-OH palmitate data, as *K*_*d*_ <P450 concentration) equations, and the *K*_*d*_ values were extracted using GraphPad Prism software (GraphPad Software, Inc).

#### Titration of Alexa-FABP1 with P450 4A11

Alexa-FABP1 (25 nM [estimated by an asborbance at 280 nm prior to conjugation reaction, accounting for dilution, and assuming no protein loss during the reaction]) was prepared in potassium phosphate buffer (10 mM or 100 mM, pH 7.4) in a 1 ml glass cuvette. A buffer baseline was recorded using an OLIS DM-45 spectrofluorometer (1.24 mm slit width) with excitation wavelength of 470 nm and scanning emission spectra from 500 to 600 nm. P450 4A11 (stored in 100 mM potassium phosphate buffer [pH 7.4] containing 20% glycerol) was titrated (0–50 nM) into the Alexa-FABP1 solution. The same experiment was repeated but with the P450 4A11 buffer used as the titrant to normalize the data for any solvent effect. Fluorescence maxima were observed at 510 nm (*F*_510_) and minima (baseline) at 600 nm (*F*_600_), and data were processed as [(*F*_510_-*F*_600_)_P450_/(*F*_510_-*F*_600_)_buffer_ −1] at each data point to yield the normalized data. As stimulation was observed by the P450 interaction, the value of one was subtracted from the normalized fluorescence values so that the curve would have an (*x*, *y*) intercept of (0, 0). *K*_*d*_ values were calculated fitting plots of the normalized fluorescence at emission maximum *versus* concentration of P450 4A11 using a quadratic equation in GraphPad Prism software.

### *k*_on_ determinations

#### P450 4A11 with lipids

On-rates (*k*_on_) to P450 4A11 with lipids were determined by monitoring the hypsochromic (“blue,” “type I”) shift in the heme Soret (*A*_418_ [hexacoordinated] → *A*_390_ [pentacoordinated]) as substrate binds.

Solutions of (A) P450 4A11 (2 μM) and (B) palmitate (10 μM) (2 ml each, all in 100 mM potassium phosphate buffer [pH 7.4]) and loaded into the drive syringes of an OLIS-RSM 1000 stopped-flow spectrophotometer (at 23 °C) with slit widths of 1.24 mm (8 nm bandpass) and set to scan from 332 to 565 nm. The system was zeroed with a buffer (above) blank, and the experiment was initiated by forcing (*via* a CO_2_ drive) equal volumes (∼0.2 ml) of each solution together into a central mixing chamber (dead time ∼5 ms), giving a twofold dilution of all reaction components. The rate of lipid binding to P450 is given by Δ*A*_390_-*A*_418_ (lipid binding [increase in *A*_390_] displaces the [sixth] axial heme ligand [H_2_O, decrease in *A*_418_]). At least six replicate measurements were collected and the traces were normalized such that the reaction started at an (*x*, *y*) intercept of (0, 0). The traces were averaged, and the resulting curve was fit to a single exponential equation in GraphPad Prism software.

#### FABP1 with DAUDA, lipids

Determination of the DAUDA on-rate (*k*_on_) to FABP1 was performed by mixing FABP1 (1 μM) and DAUDA (1–50 μM, see “Preparation of DAUDA stock solutions”) solutions (both in 100 mM potassium phosphate buffer, pH 7.4) in equal proportion (yielding a twofold dilution of all reaction components) using an OLIS stopped-flow spectrofluorometer with the parameters set as described earlier (see “P450 4A11 *k*_on_ determinations”). The DAUDA fluorophore was excited at 335 nm, and all fluorescence emissions >420 nm were monitored over time (using an Oriel 420 nm end-on filter; Oriel) (the DAUDA–FABP1 complex yields a fluorescence maximum at 482 nm, see “*K*_*d*_ determinations with FABP1”). At least six replicate measurements were recorded, the spectra were processed such that the fluorescence gain (from DAUDA binding) began at an (*x*, *y*) intercept of (0, 0), and the spectra were averaged and fit to double exponential plots in GraphPad Prism, and the rates for the fast (*k*_on_) and slow phases of binding were extracted. Then, as the first experiment was recorded by mixing equimolar (1 μM) concentrations of FABP1 and DAUDA and the binding was rapid, we approximated the binding as 2A → C (*i.e.*, mathematically equivalent to A + B → C when the depletion of B is equal to the depletion of A (77)). We plotted [FABP]^−1^ as a function of time and fit the data to a linear equation (the slope of which is *k*_on_).

The *k*_on_ determination of FABP1 with palmitate was done in the same manner as described with DAUDA but with the modification that the solutions mixed in the stopped-flow spectrofluorometer were (A) FABP1 (1 μM) and DAUDA (1 μM, aliquoted from a stock solution prepared in K_2_CO_3_ [50 mM], see “Preparation of DAUDA stock solutions”) and (B) palmitate (1–50 μM, aliquoted from a stock solution prepared in K_2_CO_3_ [50 mM]). Both mixing solutions (2 ml) were prepared in potassium phosphate buffer (100 mM, pH 7.4). The data collection, processing, and analysis proceeded *via* the same steps described earlier. The only difference in interpretation is that the signal (binding of palmitate) displaces DAUDA in the FABP1-binding site, so we observe an attenuation of fluorescence.

#### Alexa-FABP1 and P450 4A11 (association kinetics)

Mixing solutions (2 ml) of (A) Alexa-FABP1 (0.25 μM [estimated by *A*_280_ prior to conjugation reaction, accounting for dilution, and assuming no protein loss during the reaction as described earlier]) and (B) P450 4A11 (0.25 μM) were prepared in potassium phosphate buffer (100 mM, pH 7.4) and loaded into the stopped-flow spectrofluorometer described earlier. The instrument (1.24 mm slit width) was set with an excitation wavelength of 470 nm and Oriel end-on filter for emission >495 nm. The fluorescence maximum of Alexa-FABP1 is 510 nm as described earlier, and stimulation is observed upon protein–protein binding (see “Titrations of Alexa-FABP1 with P450 4A11”). At least six replicate measurements were recorded, the spectra were processed such that the fluorescence gain (from DAUDA binding) began at an (*x*, *y*) intercept of (0, 0), and the spectra were averaged. A smoothing factor of 0.4 was applied to the data (in Microsoft Excel), and the resulting curve was fit to a single exponential plot in GraphPad Prism, and the rate of association was extracted. The data were loaded into KinTek Explorer and fit to a minimal kinetic model of protein–protein association (*i.e.,* E+F↔EF), and a second-order rate constant for the association was extracted.

#### FABP1 and P450 4A11 (palmitate transfer kinetics)

To estimate the rate of transfer of palmitate from FABP1 to P450 4A11, a mixture of palmitate, FABP1, and DAUDA (0.4 μM, each component) was mixed against a solution of P450 4A11 (4 μM). All components were diluted twofold to their final concentrations in the mixing process. The experiment was performed with stopped-flow parameters identical to the experiments of FABP1 with lipids (aforementioned), and data were processed in the same manner, with the exception that a smoothing factor of 0.4 was applied to the data (in Microsoft Excel) prior to curve fitting.

### P450 4A11 *k*_off_ determination

Determination of P450 4A11 *k*_off_ rates with lipids was performed using a ketoconazole “trap” experiment by adapting previously published protocols (27, 28). Solutions of (A) ketoconazole (200 μM) and (B) P450 4A11 (4 μM) and either palmitate (20 μM) or 16-OH palmitate (8 μM) were prepared (2 ml each, all in 100 mM potassium phosphate buffer [pH 7.4]) and loaded into the drive syringes of an OLIS stopped-flow spectrophotometer with the same procedure described for the P450 4A11 *k*_on_ determination (aforementioned). The experiment was initiated by mixing equal volumes of each solution together, and the rate of ketoconazole binding to P450 is given by Δ*A*_418_-*A*_390_ (displacement of lipid [decrease in *A*_390_] because of ketoconazole binding [increase in *A*_418_]) and is a maximum value for the rate at which the P450–lipid complex dissociated (*k*_off_) (Fig. S14).

### Steady-state assays

To measure reaction rate *versus* [FABP], a reconstitution mixture of P450 4A11 (0.15 μM), NADPH–P450 reductase (0.3 μM), and 1,2-dilauroyl-*sn*-glycero-3-phosphocholine (150 μM, added from a freshly sonicated 1 mg/ml stock) was prepared and incubated in ice (10 min). Potassium phosphate buffer (100 mM, pH 7.4), DTT (1 mM, added as described earlier (44, 56)), and [1-^14^C]-palmitate (5 μM, 20 mCi/mmol [added from a 1 mM stock in C_2_H_5_OH]) were added, and the mixture was split between vials. Just prior to the reaction preincubation, FABP1 (0–5 μM) was added (diluted so the same volume of FABP1 stock solutions were added to each sample). The mixtures were preincubated (5 min, 37 °C), and P450 4A11 activity was initiated with an NADPH-regenerating system (78), the addition of which brought reactions to their final volume (0.5 ml). Reactions (5 min) were quenched *via* the addition of CH_2_Cl_2_ (5 ml), were mixed with a vortex device, and were placed on ice. After the completion of the time course, the samples were centrifuged (1000*g*, 5 min, 23 °C) to separate layers, the organic layer (4 ml) was transferred to fresh vials, and the solvent was removed under a stream of N_2_. The dried residue was resuspended in C_2_H_5_OH (0.1 ml) and transferred to autosampler vials, and an aliquot was injected (70 μl) on an Agilent 1100 Series HPLC system on a NovaPak (Waters) octadecylsilane (C_18_) 3.9 mm × 150 mm (4 μm) column (held at 23 °C) with a flow rate of 2.0 ml min^−1^ using a gradient mobile phase of (A) H_2_O and (B) CH_3_CN as follows (all in % B, v/v: 0 min, 35%; 3 min, 100%; 5 min, 100%; 6 min, 35%; and 8 min, 35%). The column eluate was mixed (1:2, v/v) with FlowLogic U scintillation cocktail (LabLogic), and radioactivity (^14^C) was detected using a β-RAM Model 5 radioflow detector (IN/US; LabLogic). Product was quantitated on the basis of its ^14^C peak area relative to the summed radioactivity of the substrate and product.

To determine the reaction rate *versus* palmitate concentration, a similar reconstitution premixture was prepared with the modification that FABP1 (5 μM) was used instead of palmitate, and the concentrations of P450 4A11 and NADPH–P450 reductase were increased to 0.3 μM and 0.6 μM, respectively. All other reaction components were kept the same. Immediately prior to the reaction preincubation, palmitate (0–5 μM, added from stock solutions prepared in 50 mM K_2_CO_3_) was added (diluted such the same volume of each stock solution was added to every sample). The mixtures were preincubated (5 min, 37 °C), and P450 4A11 activity was initiated with an NADPH-regenerating system as before, the addition of which brought reactions to their final volume (0.25 ml, reduced from before to conserve material). Reactions (10 min) were then quenched, extracted, and brought to dryness as before. The residue was dissolved in 50 μl C_2_H_5_OH, transferred to autosampler vials, and analyzed on LC–MS as described earlier (see “Quantitation of ligand-bound FABP1”) but set to operate in the negative-ion mode with the gradient mobile phase modified to (all % v/v: 0 min, 65% B; 0.5 min, 65% B; 5 min, 100% B; 7.5 min, 100% B; 7.6 min, 65% B; 9 min, 65% B).

### Kinetic modeling

Data were imported into KinTek Explorer software (version 11.01; KinTek, https://kintekcorp.com/software) (76) as Excel txt files and processed using an Apple computer (operating system 11.6.2, Apple). The kinetic modeling of a binding titration of FABP1 with palmitate (Fig. S13) as well as the txt files used for all kinetic modeling are provided in the Supporting Information.

## Data availability

Additional data are available in the Supporting Information and include key information (sequences, extinction coefficients, and quantitation protocols) and characterization of proteins (SDS-PAGE gels, catalytic activity, proteomics results, and native MS) and chemicals (NMR, mass, UV, and fluorescence spectra). Also, key txt files include the data used for calculations for *k*_transfer_ and *K*_*d*_ determinations and fitting to kinetic models.

## Supporting information

This article contains supporting information. The references used are (44, 69, 70, 79, 80, 81).

## Conflict of interest

The authors declare that they have no conflicts of interest with the contents of this article.

## Supporting information

Supporting information

**Abbreviations**
CYP4, cytochrome P450 family 4
FABP1, fatty acid binding protein
GST, glutathione-S-transferase
MS, mass spectrometry
MWCO, molecular weight cutoff
Ni–NTA, nickel–nitrilotriacetic acid
P450 (or CYP), cytochrome P450
POR, NADPH–P450 reductase

## Footnotes

1 Of note is one curious report with the heart ortholog (FABP3). Like FABP1, human heart FABP (FABP3) was also demonstrated to bind arachidonate tightly (*K*_*d*_ ∼0.05 μM) but was also observed to bind its oxygenated P450 product (ω-hydroxy arachidonic acid, 20-HETE) with relatively high affinity (*K*_*d*_ ∼0.44 μM) (40). This result was unexpected given the increased polarity of the product and the comparatively high *K*_*d*_ values they determined for similar oxygenated lipids. While not direct evidence for P450 interaction, it certainly draws attention to the possibility of association of the protein with the P450 reaction, in that it has high affinity for substrate and product for a critical reaction in eicosanoid biosynthesis.
