# Supplementary material for: Liver fatty acid binding protein FABP1 transfers substrates to cytochrome P450 4A11 for catalysis
Source: J Biol Chem. 2025 Jan 8;301(2):108168. doi: 10.1016/j.jbc.2025.108168 (PMC11847541; doi:10.1016/j.jbc.2025.108168)
Supplement: Supporting information [file mmc1.docx]

**Supporting Information**

**Liver fatty acid binding protein FABP1 transfers substrates to cytochrome P450 4A11 for catalysis**

Kevin D. McCarty and F. Peter Guengerich

Department of Biochemistry, Vanderbilt University School of Medicine, Nashville, Tennessee 37232-0146, United States

**Table of contents**

**FABP1**

1. Figure S1. FABP1 nucleic acid sequence p. S-2
2. Figure S2. FABP1-pGEX-69-2 vector map p. S-3
3. Figure S3. FABP1 amino acid sequence (after proteolytic digest) p. S-4
4. Figure S4. SDS PAGE of FABP1 p. S-4
5. Figure S5. Quantitation of fatty acids extracted from FABP1 enzyme stocks p. S-5

**Quantitation of FABP1**

1. Figure S6. Molecular weight and extinction coefficient (*ε*_280_) calculation p. S-6
2. Figure S7. Quantitation via *A*_280_ (UV spectrum) p. S-6

**11-Dansylaminoundecanoic acid (DAUDA)**

1. Figure S8. DAUDA synthetic scheme p. S-7
2. Figure S9. ^1^H spectrum p. S-7
3. Figure S10. ^13^C NMR spectrum p. S-8
4. Figure S11. LC-MS & LC-UV analysis, absorbance & fluorescence spectra p. S-9

**Determination of lipid dissociation and rate constants**

1. Figure S12. Data processing for FABP1 titration with palmitate p. S-10
2. Figure S13. *K*_d_ modeling in KinTek Explorer (FABP1 + palmitate) p. S-11
3. Figure S14. Absorbance traces used to determine P450 4A11 palmitate *k*_off_ p. S-12

**Alexa-FABP1**

1. Figure S15. Spectral properties of Alexa-FABP1 p. S-12
2. Figure S16. Structure of FABP1 highlighting Cys-69 p. S-13
3. Figure S17. LC-MS/MS analysis of FABP1 Cys-69(71)-containing peptide p. S-14
4. Figure S18. Native MS of Alexa-488 labeled FABP1 conjugate. p. S-15

**P450 4A11**

1. Figure S19. Titration of P450 4A11 with DAUDA p. S-16
2. Figure S20. Catalytic activity assay ± cytochrome *b*_5_ p. S-16
3. Figure S21. Reduced CO difference spectra p. S-17

**Kinetic analysis (txt files input into modeling software)**

1. *K*_d_ determinations p. S-17
2. Steady-state reactions (P450 4A11 + palmitate + FABP1) p. S-19

**FABP1**

ACGTTATCGACTGCACGGTGCACCAATGCTTCTGGCGTCAGGCAGCCATCGGAAGCTGTGGTATGGCTGTGCAGGTCGTAAATCACTGCATAATTCGTGTCGCTCAAGGCGCACTCCCGTTCTGGATAATGTTTTTTGCGCCGACATCATAACGGTTCTGGCAAATATTCTGAAATGAGCTGTTGACAATTAATCATCGGCTCGTATAATGTGTGGAATTGTGAGCGGATAACAATTTCACACAGGAAACAGTATTCATGTCCCCTATACTAGGTTATTGGAAAATTAAGGGCCTTGTGCAACCCACTCGACTTCTTTTGGAATATCTTGAAGAAAAATATGAAGAGCATTTGTATGAGCGCGATGAAGGTGATAAATGGCGAAACAAAAAGTTTGAATTGGGTTTGGAGTTTCCCAATCTTCCTTATTATATTGATGGTGATGTTAAATTAACACAGTCTATGGCCATCATACGTTATATAGCTGACAAGCACAACATGTTGGGTGGTTGTCCAAAAGAGCGTGCAGAGATTTCAATGCTTGAAGGAGCGGTTTTGGATATTAGATACGGTGTTTCGAGAATTGCATATAGTAAAGACTTTGAAACTCTCAAAGTTGATTTTCTTAGCAAGCTACCTGAAATGCTGAAAATGTTCGAAGATCGTTTATGTCATAAAACATATTTAAATGGTGATCATGTAACCCATCCTGACTTCATGTTGTATGACGCTCTTGATGTTGTTTTATACATGGACCCAATGTGCCTGGATGCGTTCCCAAAATTAGTTTGTTTTAAAAAACGTATTGAAGCTATCCCACAAATTGATAAGTACTTGAAATCCAGCAAGTATATAGCATGGCCTTTGCAGGGCTGGCAAGCCACGTTTGGTGGTGGCGACCATCCTCCAAAATCGGATCTGGAAGTTCTGTTCCAGGGGCCCATGAGTTTTTCAGGAAAATATCAGCTACAATCCCAAGAGAACTTCGAGGCATTCATGAAAGCTATCGGTCTGCCGGAGGAGCTGATTCAGAAAGGTAAGGATATCAAAGGCGTTAGCGAAATTGTGCAGAACGGCAAGCACTTTAAGTTCACCATCACTGCGGGTAGCAAGGTGATTCAAAACGAATTCACCGTTGGTGAGGAGTGCGAATTGGAAACCATGACCGGTGAAAAAGTGAAAACCGTCGTCCAGCTGGAGGGCGATAATAAGCTGGTTACGACCTTTAAGAACATTAAATCTGTTACGGAATTGAATGGCGACATCATCACCAATACCATGACGCTGGGCGACATCGTGTTTAAGCGCATCAGCAAACGTATTCACCACCACCACCACCACTAATGACTGACTGACGATCTGCCTCGCGCGTTTCGGTGATGACGGTGAAAACCTCTGACACATGCAGCTCCCGGAGACGGTCACAGCTTGTCTGTAAGCGGATGCCGGGAGCAGACAAGCCCGTCAGGGCGCGTCAGCGGGTGTTGGCGGGTGTCGGGGCGCAGCCATGACCCAGTCACGTAGCGATAGCGGAGTGTATAATTCTTGAAGACGAAAGGGCCTCGTGATACGCCTATTTTTATAGGTTAATGTCATGATAATAATGGTTTCTTAGACGTCAGGTGGCACTTTTCGGGGAAATGTGCGCGGAACCCCTATTTGTTTATTTTTCTAAATACATTCAAATATGTATCCGCTCATGAGACAATAACCCTGATAAATGCTTCAATAATATTGAAAAAGGAAGAGTATGAGTATTCAACATTTCCGTGTCGCCCTTATTCCCTTTTTTGCGGCATTTTGCCTTCCTGTTTTTGCTCACCCAGAAACGCTGGTGAAAGTAAAAGATGCTGAAGATCAGTTGGGTGCACGAGTGGGTTACATCGAACTGGATCTCAACAGCGGTAAGATCCTTGAGAGTTTTCGCCCCGAAGAACGTTTTCCAATGATGAGCACTTTTAAAGTTCTGCTATGTGGCGCGGTATTATCCCGTGTTGACGCCGGGCAAGAGCAACTCGGTCGCCGCATACACTATTCTCAGAATGACTTGGTTGAGTACTCACCAGTCACAGAAAAGCATCTTACGGATGGCATGACAGTAAGAGAATTATGCAGTGCTGCCATAACCATGAGTGATAACACTGCGGCCAACTTACTTCTGACAACGATCGGAGGACCGAAGGAGCTAACCGCTTTTTTGCACAACATGGGGGATCATGTAACTCGCCTTGATCGTTGGGAACCGGAGCTGAATGAAGCCATACCAAACGACGAGCGTGACACCACGATGCCTGCAGCAATGGCAACAACGTTGCGCAAACTATTAACTGGCGAACTACTTACTCTAGCTTCCCGGCAACAATTAATAGACTGGATGGAGGCGGATAAAGTTGCAGGACCACTTCTGCGCTCGGCCCTTCCGGCTGGCTGGTTTATTGCTGATAAATCTGGAGCCGGTGAGCGTGGGTCTCGCGGTATCATTGCAGCACTGGGGCCAGATGGTAAGCCCTCCCGTATCGTAGTTATCTACACGACGGGGAGTCAGGCAACTATGGATGAACGAAATAGACAGATCGCTGAGATAGGTGCCTCACTGATTAAGCATTGGTAACTGTCAGACCAAGTTTACTCATATATACTTTAGATTGATTTAAAACTTCATTTTTAATTTAAAAGGATCTAGGTGAAGATCCTTTTTGATAATCTCATGACCAAAATCCCTTAACGTGAGTTTTCGTTCCACTGAGCGTCAGACCCCGTAGAAAAGATCAAAGGATCTTCTTGAGATCCTTTTTTTCTGCGCGTAATCTGCTGCTTGCAAACAAAAAAACCACCGCTACCAGCGGTGGTTTGTTTGCCGGATCAAGAGCTACCAACTCTTTTTCCGAAGGTAACTGGCTTCAGCAGAGCGCAGATACCAAATACTGTCCTTCTAGTGTAGCCGTAGTTAGGCCACCACTTCAAGAACTCTGTAGCACCGCCTACATACCTCGCTCTGCTAATCCTGTTACCAGTGGCTGCTGCCAGTGGCGATAAGTCGTGTCTTACCGGGTTGGACTCAAGACGATAGTTACCGGATAAGGCGCAGCGGTCGGGCTGAACGGGGGGTTCGTGCACACAGCCCAGCTTGGAGCGAACGACCTACACCGAACTGAGATACCTACAGCGTGAGCTATGAGAAAGCGCCACGCTTCCCGAAGGGAGAAAGGCGGACAGGTATCCGGTAAGCGGCAGGGTCGGAACAGGAGAGCGCACGAGGGAGCTTCCAGGGGGAAACGCCTGGTATCTTTATAGTCCTGTCGGGTTTCGCCACCTCTGACTTGAGCGTCGATTTTTGTGATGCTCGTCAGGGGGGCGGAGCCTATGGAAAAACGCCAGCAACGCGGCCTTTTTACGGTTCCTGGCCTTTTGCTGGCCTTTTGCTCACATGTTCTTTCCTGCGTTATCCCCTGATTCTGTGGATAACCGTATTACCGCCTTTGAGTGAGCTGATACCGCTCGCCGCAGCCGAACGACCGAGCGCAGCGAGTCAGTGAGCGAGGAAGCGGAAGAGCGCCTGATGCGGTATTTTCTCCTTACGCATCTGTGCGGTATTTCACACCGCATAAATTCCGACACCATCGAATGGTGCAAAACCTTTCGCGGTATGGCATGATAGCGCCCGGAAGAGAGTCAATTCAGGGTGGTGAATGTGAAACCAGTAACGTTATACGATGTCGCAGAGTATGCCGGTGTCTCTTATCAGACCGTTTCCCGCGTGGTGAACCAGGCCAGCCACGTTTCTGCGAAAACGCGGGAAAAAGTGGAAGCGGCGATGGCGGAGCTGAATTACATTCCCAACCGCGTGGCACAACAACTGGCGGGCAAACAGTCGTTGCTGATTGGCGTTGCCACCTCCAGTCTGGCCCTGCACGCGCCGTCGCAAATTGTCGCGGCGATTAAATCTCGCGCCGATCAACTGGGTGCCAGCGTGGTGGTGTCGATGGTAGAACGAAGCGGCGTCGAAGCCTGTAAAGCGGCGGTGCACAATCTTCTCGCGCAACGCGTCAGTGGGCTGATCATTAACTATCCGCTGGATGACCAGGATGCCATTGCTGTGGAAGCTGCCTGCACTAATGTTCCGGCGTTATTTCTTGATGTCTCTGACCAGACACCCATCAACAGTATTATTTTCTCCCATGAAGACGGTACGCGACTGGGCGTGGAGCATCTGGTCGCATTGGGTCACCAGCAAATCGCGCTGTTAGCGGGCCCATTAAGTTCTGTCTCGGCGCGTCTGCGTCTGGCTGGCTGGCATAAATATCTCACTCGCAATCAAATTCAGCCGATAGCGGAACGGGAAGGCGACTGGAGTGCCATGTCCGGTTTTCAACAAACCATGCAAATGCTGAATGAGGGCATCGTTCCCACTGCGATGCTGGTTGCCAACGATCAGATGGCGCTGGGCGCAATGCGCGCCATTACCGAGTCCGGGCTGCGCGTTGGTGCGGATATCTCGGTAGTGGGATACGACGATACCGAAGACAGCTCATGTTATATCCCGCCGTCAACCACCATCAAACAGGATTTTCGCCTGCTGGGGCAAACCAGCGTGGACCGCTTGCTGCAACTCTCTCAGGGCCAGGCGGTGAAGGGCAATCAGCTGTTGCCCGTCTCACTGGTGAAAAGAAAAACCACCCTGGCGCCCAATACGCAAACCGCCTCTCCCCGCGCGTTGGCCGATTCATTAATGCAGCTGGCACGACAGGTTTCCCGACTGGAAAGCGGGCAGTGAGCGCAACGCAATTAATGTGAGTTAGCTCACTCATTAGGCACCCCAGGCTTTACACTTTATGCTTCCGGCTCGTATGTTGTGTGGAATTGTGAGCGGATAACAATTTCACACAGGAAACAGCTATGACCATGATTACGGATTCACTGGCCGTCGTTTTACAACGTCGTGACTGGGAAAACCCTGGCGTTACCCAACTTAATCGCCTTGCAGCACATCCCCCTTTCGCCAGCTGGCGTAATAGCGAAGAGGCCCGCACCGATCGCCCTTCCCAACAGTTGCGCAGCCTGAATGGCGAATGGCGCTTTGCCTGGTTTCCGGCACCAGAAGCGGTGCCGGAAAGCTGGCTGGAGTGCGATCTTCCTGAGGCCGATACTGTCGTCGTCCCCTCAAACTGGCAGATGCACGGTTACGATGCGCCCATCTACACCAACGTAACCTATCCCATTACGGTCAATCCGCCGTTTGTTCCCACGGAGAATCCGACGGGTTGTTACTCGCTCACATTTAATGTTGATGAAAGCTGGCTACAGGAAGGCCAGACGCGAATTATTTTTGATGGCGTTGGAATT

**Figure S1.** FABP1 nucleic acid sequence.

**
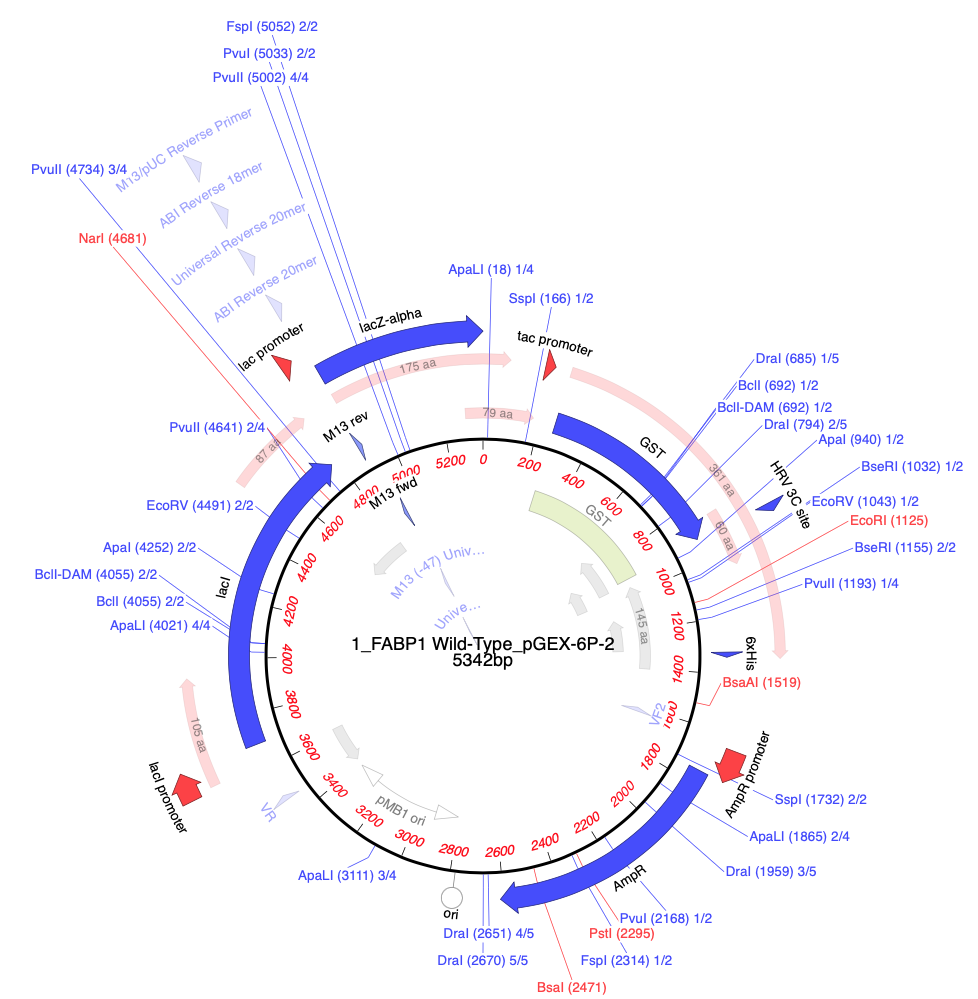
**

**Figure S2.** FABP1-pGEX-69-2 vector map. The nucleotide sequence of the FABP1 insert is shown in Fig. S1.

10 20 30 40 50 60
**GP**MSFSGKYQ LQSQENFEAF MKAIGLPEEL IQKGKDIKGV SEIVQNGKHF KFTITAGSKV

 70 80 90 100 110 120
IQNEFTVGEE CELETMTGEK VKTVVQLEGD NKLVTTFKNI KSVTELNGDI ITNTMTLGDI

 130
VFKRISKRI**H HHHHH**

**Figure S3.** FABP1 amino acid sequence (after proteolytic digest). Note: As indicated by the manufacturer, the optimum recognition site of PreScission Protease is the sequence Leu-Glu-Val-Leu-Phe-Gln/Gly-Pro (LEVLFQ/GP) and cleavage occurs between the Gln and Gly-Pro residues. Consequently, digestion of GST-tagged FABP1 leaves two amino acids at the N-terminus of WT FABP1: the residues **GP**. A C-terminal **hexahistidine tag** in the FABP1 was also included to the construct to facilitate purification by metal ion affinity chromatography. All residues (tags) not native to the 127 amino acid WT protein are underlined and bolded for clarity.

**
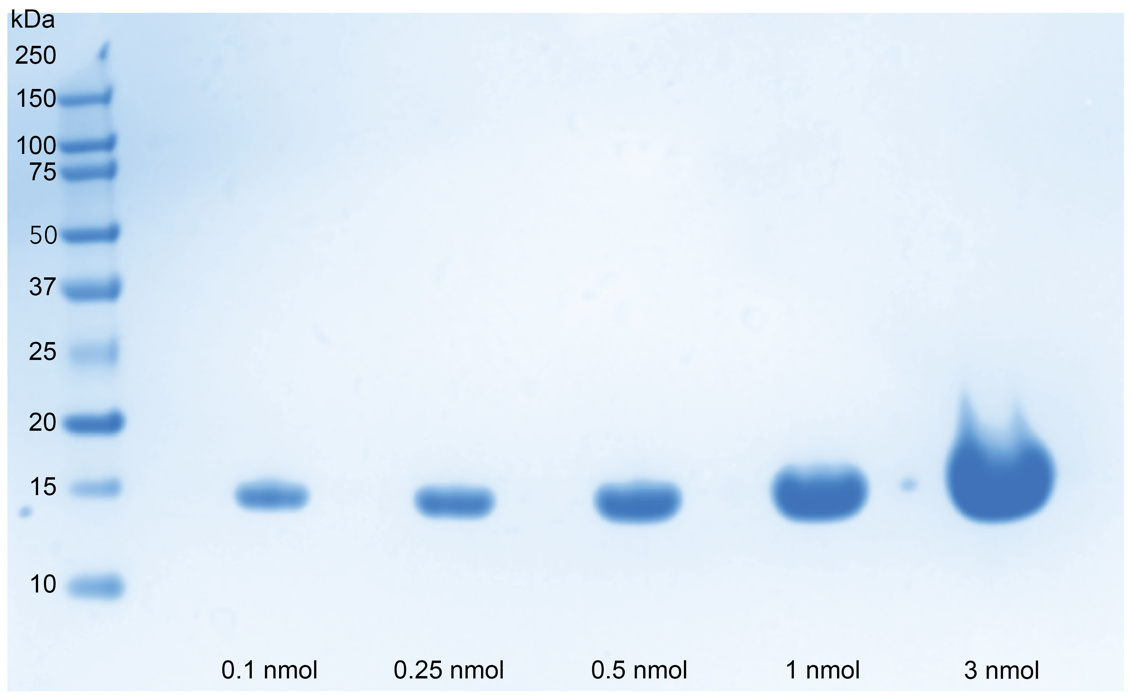
**

**Figure S4. Electrophoretic purity of recombinant FABP1.** SDS-polyacrylamide gel electrophoresis (2-(*N*-morpholino)ethanesulfonic acid (MES) SDS running buffer, 150 V, 60 min) was done using a 10% Bis-Tris gel and stained with Coomassie blue. MW markers (Precision Plus Protein Kaleidoscope^TM^) and protein load are labeled.

**Figure S5. Quantitation of fatty acids extracted from FABP1 enzyme stocks.** *A,* quantitation of total fatty acids (FA, C_12_-C_18_) detected from a water blank, FABP1 prior to delipidation (0X Lipidex) and after delipidation using one incubation (30 min, 23 °C) with Lipidex (1× Lipidex), two incubations of Lipidex (2× Lipdex), or delipidation with three extractions with an equal volume of 1-butanol followed by one incubation with Lipidex (Butanol, Lipidex). Fatty acid extraction, derivatization, and detection were performed as described in the Materials and Methods. *B,* same as in *A*, except that the data was normalized to the solvent control such that only the fatty acid content of the FABP1 is quantified. Fatty acids were detected as pyridyl ester derivatives utilizing positive ion ESI LC-MS as described in the Materials and Methods (See “Quantitation of ligand-bound FABP1”).

**Quantitation of FABP1**


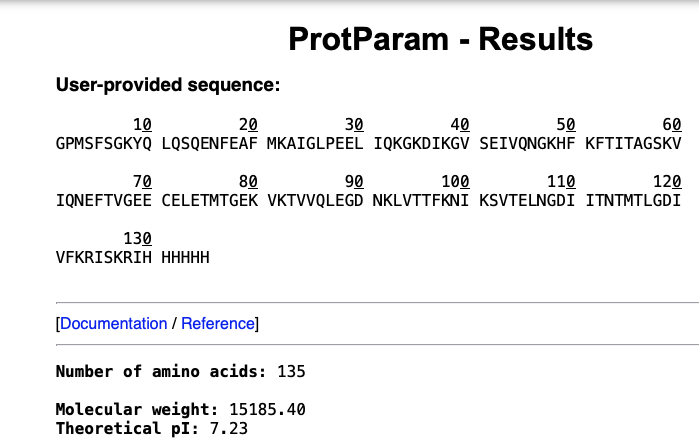


**
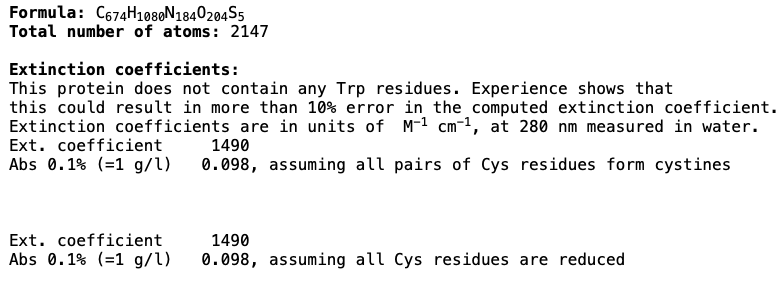
**

**Figure S6. FABP1 molecular weight and molar extinction coefficient.** The molecular weight and molar extinction coefficient of FABP1 (at 280 nm (*ε*_280_)) was estimated theoretically using the ExPASY ProtParam tool (70). Shown above is a screenshot of the output from the software when the FABP1 sequence provided in Fig. S3 is entered as the input. The predicted molecular weight is 15185.40 Da (~15.2 kDa) and the predicted *ε*_280_ is 1490 M^-1^ cm^-1^. The predicted ε*_2_*_80_ gives the same result as the general approach of *ε*_280_ (in M^-1^ cm^-1^) = 1480*(n_tyrosine_) + 5540*(n_tryptophan_) (78) as FABP1 has one tyrosine and zero tryptophan residues (giving an estimate of 1480 M^-1^ cm^-1^). The computational estimate of 1490 M^-1^ cm^-1^ was used in our work.

**Figure S7. FABP1 quantitation via *A*_280_ (UV spectrum).** UV spectra were collected in 1 cm quartz microcuvettes (0.35 mL) using an OLIS-Cary 14 spectrophotometer (On-Line Instrument Systems, Athens, GA) scanning from 250-400 nm. A baseline was recorded in 100 mM potassium phosphate buffer (pH 7.4) containing 100 mM NaCl. Three absorbance spectra of the purified protein stock were then collected and averaged. The absorbances at 280 nm (dotted line) and 398 nm (baseline) were recorded and the difference was taken (*A*_280_-*A*_398_) and divided by the extinction coefficient estimated for FABP1 earlier (i.e., Fig. S6, 1490 M^-1^ cm^-1^). The protein stock in the spectrum above was calculated to be 169 µM.

**11-Dansylaminoundecanoic acid (DAUDA)**

**Figure S8.** DAUDA synthetic scheme.

**Figure S9. ^1^H NMR spectrum of DAUDA.**

**Figure S10. ^13^C NMR spectrum of DAUDA.**

**Fig S11. Analysis of synthetic DAUDA.** *A*, LC-MS analysis of DAUDA using a Waters QDa single quadrupole mass spectrometer operating in the positive ion mode. Only one peak (*t*_R_ 9.1 min) was visible in the total ion current (TIC) channel (excluding the noise at the time of injection). *B*, LC-UV analysis of DAUDA Waters photodiode array detector. *C*, mass spectrum (150-500 *m/z*) of the peak (*t*_R_ 9.1 min) in part *A* showing predominantly the [MH]^+^ parent ion (*m/z* 435.3). Inset: only the spectral range from *m/z* 400-500 is shown to demonstrate the DAUDA Na^+^ and K^+^ salt adducts identified (*m/z* 457.2 and 473.2, respectively). *D*, UV spectrum of DAUDA (100 µM) measured in C_2_H_5_OH. The calculated molar extinction coefficient at 335 nm (*ε*_335_) was 5060 M^-1^ cm^-1^. *E*, Fluorescence spectrum of increasing concentrations (**—** buffer, **—** 0.1 µM, **—** 0.5 µM, and **—** 1 µM) of DAUDA in 100 mM potassium phosphate buffer (pH 7.4) with an excitation wavelength of 335 nm and scanning emission wavelength from 400-600 nm.

**Substrate binding and kinetic assays**

**Figure S12. Data processing for FABP1 titration with palmitate.** Fluorescence spectrum of increasing additions of (*A*) palmitate (0-2 µM, added in C_2_H_5_OH) and (*B*) C_2_H_5_OH to a mixture of DAUDA (0.25 µM) and FABP1 (0.5 µM), performed as described in the Experimental Methods (see “Titrations of FABP1 with lipids, DAUDA”). *C,* fluorescence values at 482 nm (F_482_) from the titrations presented in parts *A* and *B* are normalized to the starting values (DAUDA-FABP1 complex, no volume added). Data are reported as a function of volume of titrant added to the cuvette (black, C_2_H_5_OH alone; red, palmitate in C_2_H_5_OH). Although the solvent alone (C_2_H_5_OH) results in gradual attenuation of fluorescence, the addition of palmitate (up to 2 µM) sharply reduces the F_482_ signal via DAUDA displacement. *D,* the displacement of DAUDA by palmitate is shown as a function of palmitate concentration (i.e., same as *C* but as a function of concentration). *E,* the attenuation of F_482_ by palmitate (in C_2_H_5_OH) is normalized to the attenuation of F_482_ by C_2_H_5_OH alone. The data presented in part *E* represents the final step of the data processing for the DAUDA-FABP1 equilibrium binding titrations. These data were subsequently imported into KinTek Explorer for fitting to the kinetic model (Fig. S13).

**
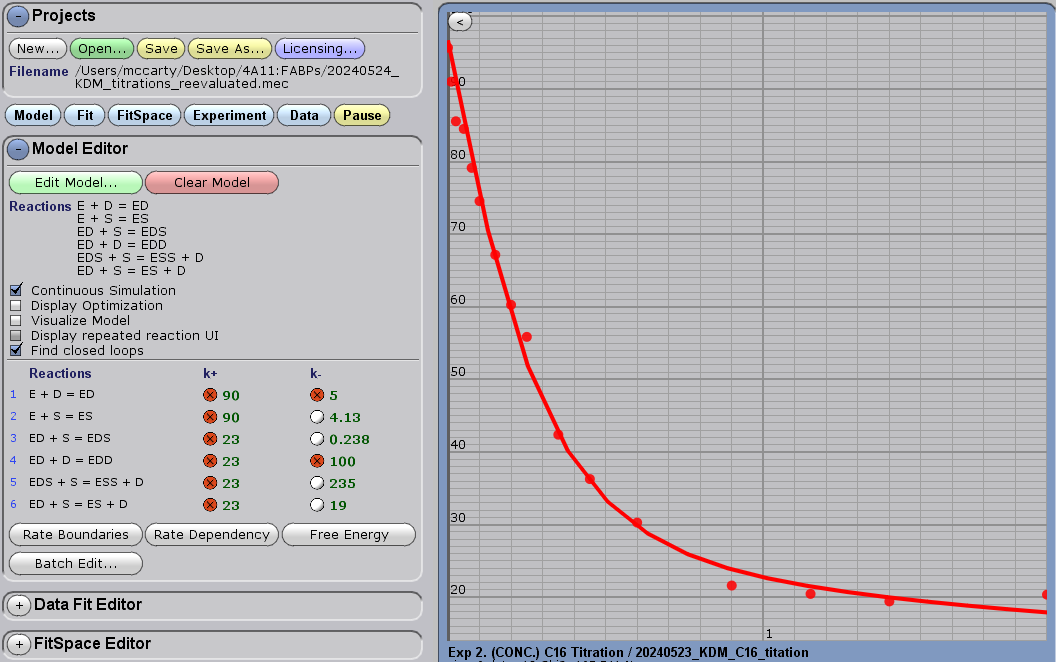
**

**Figure S13. *K*_d_ modeling in KinTek Explorer (FABP1 + palmitate).** The kinetic data from Fig. S12*E* were fit to the kinetic model shown above (under “Reactions”). The modeling was performed as described in the Experimental Methods. All rate constants filled with an **×** (at left) were pre-set in the model and locked (unable to be adjusted by the modeling program), while the open circles indicate rate constants that were fit by the model. The experimental data are shown in the curve at right (red dots), with the modeled curve (red line) superimposed. The rate constants that were fit by the model were then used to estimate the dissociation constants of FABP1 with lipids (Table 1). Software was programmed as described (75).

**Figure S14. Absorbance traces used to determine P450 4A11 palmitate *k*_off_ rate.** The basis of the ketoconazole trap experiment is the spectral transition from substrate-bound (“Type I” shift, *A*_390_ (blue trace)) to inhibitor-bound (“Type II” shift, *A*_418_ (red trace)) P450 (Fig. 9*A*). The formation of ketoconazole-bound P450 (inhibitor complex) from palmitate-bound P450 (substrate complex) is over the course of the experiment shown above. The rate of formation of inhibitor-bound P450 is subtracted from the rate of decay of substrate-bound P450, and the resulting plot (Fig. 9*D*) is fit to a biexponential equation to estimate the dissociation rate (*k*_off_) of palmitate from the P450 4A11.

**Alexa-FABP1**

**Figure S15. Spectral properties of Alexa-488 FABP1.** *A.* Absorbance spectrum (220-600 nm) of Alexa 488-labeled FABP1 (~19 µM). *B,* fluorescence emission spectrum of Alexa-FABP1 (500-600 nm) (excitation at 493 nm).

**
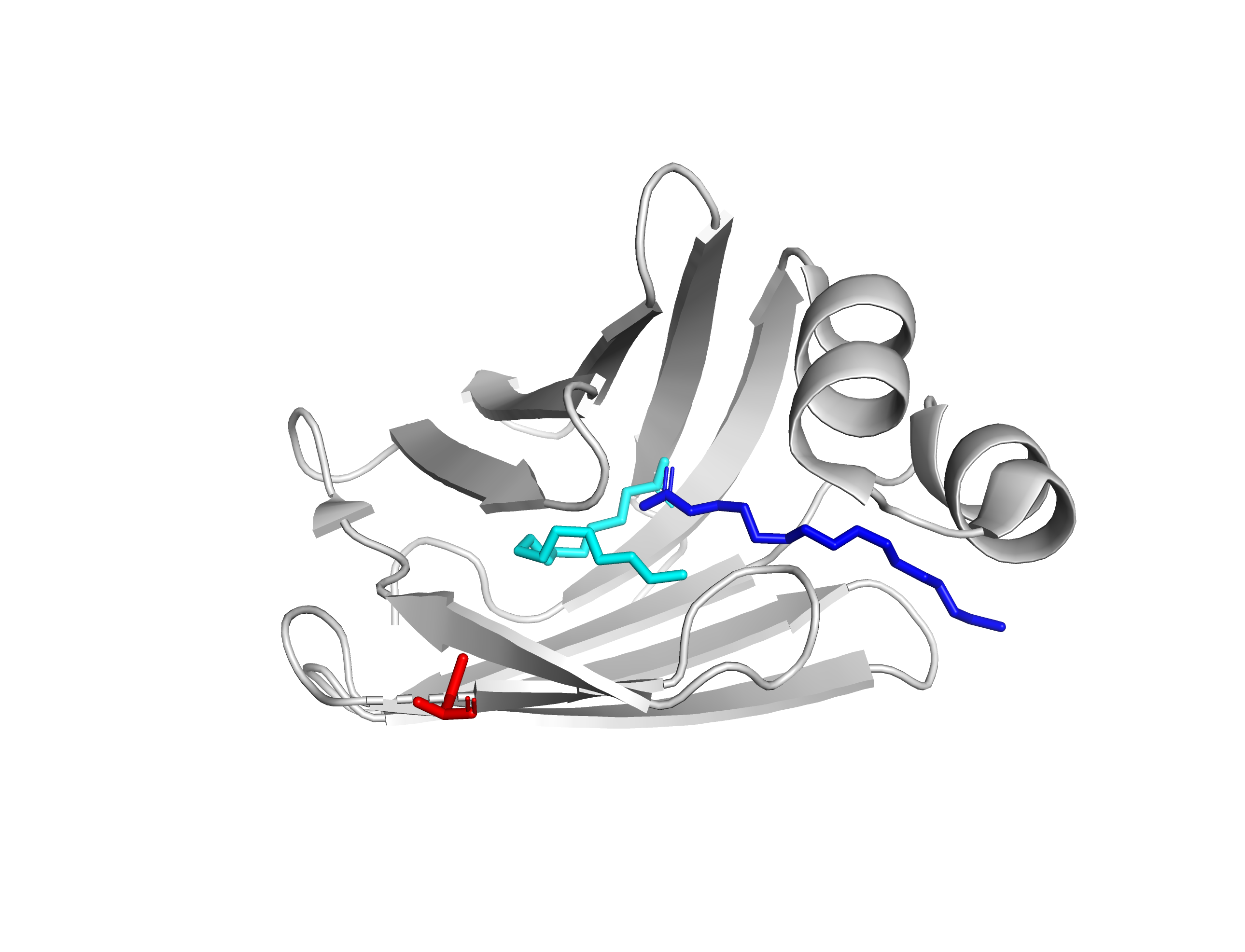
**

**Figure S16. Structure of FABP1 highlighting Cys-69.** FABP1 (gray ribbon structure) with two molecules of palmitate (teal and dark blue) bound in the binding site (PDB ID: 3STK) was selected to indicate the location of Cys-69 (structure in gray, residue in red) in the FABP1 tertiary structure relative to the palmitate binding sites. Note: Cys-69 in the WT protein structure corresponds to Cys-71 in our construct due to the residual glycine and proline residues left at the N-terminus of our construct resulting from the glutathione transferase tag after proteolytic digestion (Experimental Methods, Fig. S3).

**
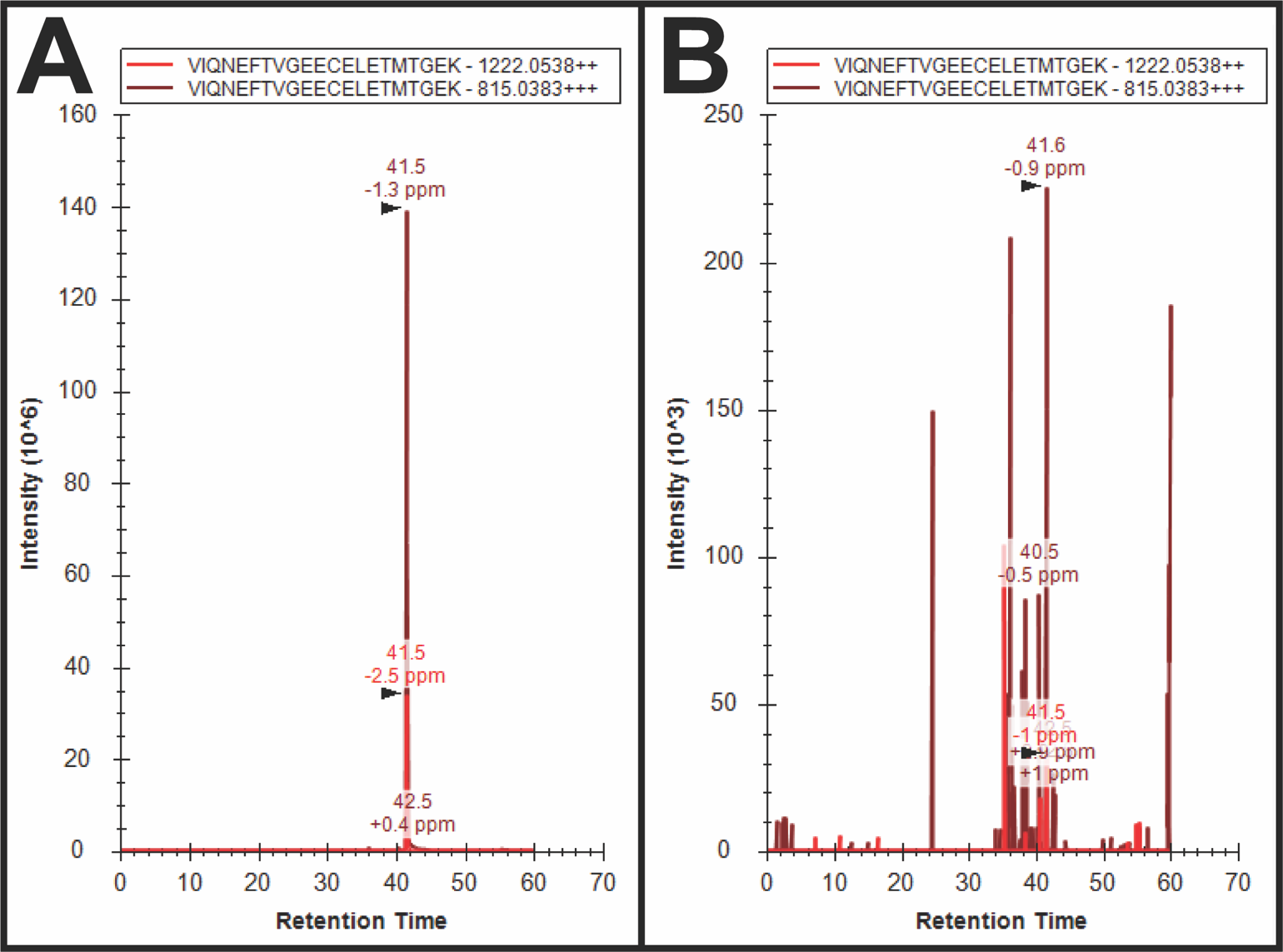
**

**Figure S17. LC-MS/MS analysis of FABP1 Cys-69(71)-containing peptides.** Solutions of FABP1 (10 µM) and Alexa-FABP1 (10 µM) were subjected to tryptic digestion and LC-MS/MS analysis as described in the Experimental Methods (see “Analysis of FABP1 peptides”). The tryptic peptide VIQNEFTVGEE**C**ELETMTGEK contains Cys-69 (in bold (Cys-71 in our construct, see Fig. S3)). When the peptides were reduced (with DTT) and treated with iodoacetamide (according to standard procedure), alkylation adducts (carbamidomethyl, of the Cys-71 residue) were primarily observed on (A) the unmodified FABP1 peptide. The 2+ (++, red trace) and 3+ (+++, black trace) charge states of the peptide are shown. The Alexa Flor 488 treated FABP1 sample (B) yielded an Alexa-FABP1 peptide that was alkylated ~850-fold less (based on comparison of peak areas of 963,800,576 (unmodified) vs. 1,137,880 (Alexa-modified) integrated in the 3+ charge state)) than the unmodified sample, indicating that the Cys-71 residue was almost entirely unavailable for alkylation in our Alexa-FABP1 protein solution (as it was already conjugated with Alexa Fluor-488 dye). This indicates that the labeling technique employed with Alexa Flor-488 dye (Experimental Methods) was highly effective at labeling Cys-71 of the FABP1 construct.

**
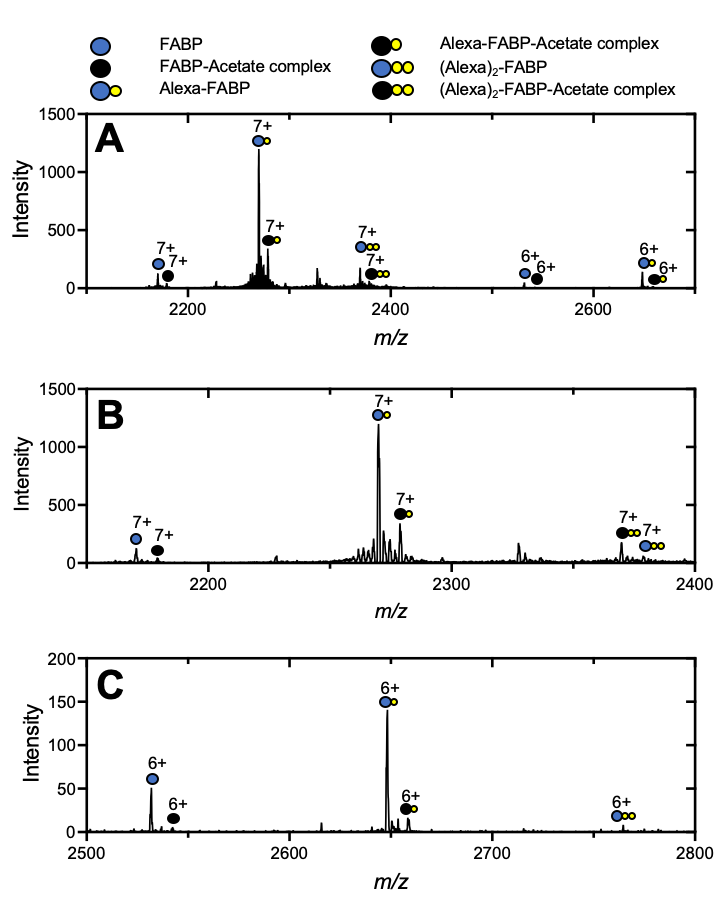
Figure S18. Native mass spectrometry of Alexa-488 labeled FABP1 conjugate.** *A*, spectrum acquired from *m/z* 2100 to 2700 showing FABP1 (15185.4 Da, Figs. S3 & S6) ionized in the 6+ and 7+ charge states. Both unlabeled (not reacted with Alexa-488 dye) and labeled (reacted with Alexa-488 dye, yellow circle) FABP1 (blue circle) were identified in each charge state. Acetate-liganded FABP1 (black circle) was also identified across all charge states (as we observed previously, Fig. 2). *B*, same is in part *A*, but only peaks corresponding to FABP1 7+ charge state (*m/z* 2100 to 2400) are shown. *C*, same as in *A,* but only peaks corresponding to FABP1 6+ charge state (*m/z* 2500 to 2800) are shown.

**Figure S19. Titration of P450 4A11 with DAUDA.** The P450 concentration was 1 µM and the DAUDA concentration was varied using a geometric series from 1 nM to 10 µM. The typical hypsochromic (“blue”, “Type I”) spectral shift (i.e. to ~390 nm) when many substrates are added to P450s was not detected upon the addition of DAUDA to P450 4A11. The increase in absorbance in the UV region is due to the strong chromophore in the DAUDA dansyl moiety (Fig. S8).

**Figure S20. Catalytic activity assay of P450 4A11 ± cytochrome *b*_5_.** P450 4A11 (0.05 µM) and NADPH-P450 reductase (0.1 µM) (± cytochrome *b*_5_, 0.1 µM) were reconstituted in DLPC (150 µM) as described in the Materials and Methods (see “Steady-state assays”). Neither plot was observed to saturate at the conditions tested; estimates for *k*_cat_ were 59 ± 3 min^-1^ (+ *b*_5_) and 29 ± 3 min^-1^ (- *b*_5_), though the maximal observed rate of reaction was 12 ± 1 min^-1^ (- *b*_5_). Accordingly, for reactions performed in the absence of *b*_5_ a maximum rate of 0.2 s^-1^ was used as a starting value in the kinetic modeling. The estimation of *k*_cat_ was done by fitting to *k*_cat_/*K*_m_ as described (80). (The ~2-fold stimulation of P450 4A11 ω-oxidation observed here was also observed with lauric acid as the substrate (44))

**Figure S21. P450 4A11 reduced-CO vs. reduced spectra.** A P450 4A11 enzyme stock was diluted (50-fold) in buffer (100 mM potassium phosphate buffer containing 20% glycerol), and the spectrophotometer was zeroed and a baseline was recorded (in black). The sample was bubbled with CO (60 s) and reduced with dithionite as described previously (81) using the extinction coefficient (*ε*_450-490_) of 91 mM^-1^ cm^-1^ (69). Safranin T dye (~4 µM) was added to facilitate the reduction. Five spectra were acquired in succession. The P450 band reached a maximum and was stable and no significant P420 band (inactive protein) was observed. The protein concentration was 0.83 µM in the cuvette.

**Kinetic analysis (txt files input into modeling software)**

***K*_d_ determinations**

FABP1 with Laurate

µM % Fluorescence

0 99.4612475

1 94.9487155

2 89.7726398

3.5 89.0302718

5 85.8930273

7.5 85.6856144

10 75.2950881

20 57.4652293

35 47.2740101

50 41.274787

75 35.154576

100 31.3348629

125 28.582828

150 29.6518861

200 27.6141368

300 25.7445275

400 23.1518994

FABP1 with Myristate

µM % Fluorescence

0 99.256

0.01 98.345

0.03 99.124

0.06 98.231

0.1 97.334

0.3 92.888

0.6 81.015

1 71.884

3 42.201

6 25.853

10 18.234

30 13.067

FABP1 with Palmitate

µM % Fluorescence

0 95.67213754

0.01 90.99583745

0.025 85.53791248

0.05 84.48819708

0.075 79.12241782

0.1 74.5457751

0.15 67.15053141

0.2 60.26117183

0.25 55.84991092

0.35 42.38033681

0.45 36.27020155

0.6 30.27650607

0.9 21.60690275

1.15 20.45628153

1.4 19.42265769

1.9 20.35329973

FABP1 with Stearate

µM % Fluorescence

0 100.5006244

0.01 92.25134217

0.025 92.50423914

0.05 87.57085264

0.075 81.32268584

0.1 78.30500759

0.15 69.44269731

0.2 60.96918998

0.25 54.17207223

0.35 44.41091635

0.45 37.11768982

0.6 31.29838317

0.9 24.17933854

1.15 24.10277377

1.4 23.58770986

1.9 23.26300654

**Steady state reaction** (Fig. 12A, [Product] vs. [FABP1], 5 µM substrate)

[FABP1], µM [P], µM

0 0.818983364

0.05 0.815209275

0.15 0.76926289

0.3 0.789661888

0.6 0.785073944

1 0.723192327

1.5 0.778024484

2.5 0.727624591

5 0.677313277

**Steady state reaction** (Fig. 12B, [Product] vs. [Substrate] – no FABP1)

[S], µM [P], µM

0 0

0.5 0.034452

0.75 0.0710475

1 0.079056

1.5 0.097446

2 0.1926225

3 0.1935795

4 0.2343255

5 0.3243075

**Steady state reaction** (Fig. 12B, [Product] vs. [Substrate], 5 µM FABP1)

[S], µM [P], µM

0 0

0.5 0.007728

0.75 0.0262425

1 0.0544395

1.5 0.0868785

2 0.1059165

3 0.146157

4 0.1710345

5 0.1794255
